# Highly Frustrated Poly(ionic liquid) ABC Triblock Terpolymers with Exceptionally High Morphology Factors

**DOI:** 10.1021/acs.macromol.3c02435

**Published:** 2024-04-02

**Authors:** Patrick M. Lathrop, Rui Sun, Frederick L. Beyer, Yossef A. Elabd

**Affiliations:** † Department of Chemical Engineering, Texas A&M University, College Station, Texas 77843, United States; ‡ U.S. Army Research Laboratory, Aberdeen Proving Ground, Maryland 21005, United States

## Abstract

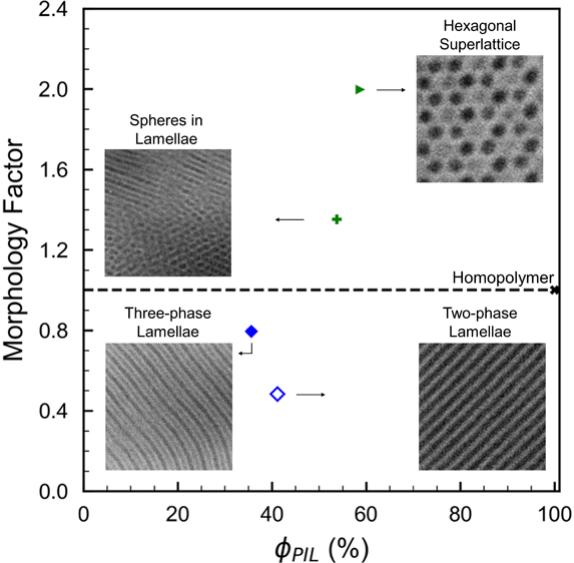

In this work, we report the successful synthesis of 17 unique compositions of a poly(ionic liquid) (PIL) ABC triblock terpolymer, poly(S-*b*-VBMIm-TFSI-*b*-HA), where S is styrene, VBMIm-TFSI is vinylbenzyl methylimidazolium bis(trifluoromethanesulfonyl)imide, and HA is hexyl acrylate. Nine distinct morphologies were observed, including two-phase and three-phase disordered microphase separated (D_2_ and D_3_), two-phase hexagonally packed cylinders (C_2_), core–shell hexagonally packed cylinders (C_CS_), three-phase lamellae (L_3_), two-phase lamellae (L_2_), core–shell double gyroid (Q^230^), spheres-in-lamellae (L_SI_), and a three-phase hexagonal superlattice of cylinders (C_SL_). The L_SI_ morphology was unambiguously confirmed using small-angle X-ray scattering and transmission electron microscopy. Morphology type significantly impacted the ion conductivity of the PIL ABC triblock terpolymers, where remarkable changes in morphology factor (normalized ion conductivity) were observed with only small changes in the conducting volume fraction, i.e., PIL block composition. An exceptionally high morphology factor of 2.0 was observed from the PIL ABC triblock terpolymer with a hexagonal superlattice morphology due to the three-dimensional narrow, continuous PIL nanodomains that accelerate ion conduction. Overall, this work demonstrates the first systematic study of highly frustrated single-ion conducting ABC triblock terpolymers with a diverse set of morphologies and exceptionally high morphology factors, enabling the exploration of transport–morphology relationships to guide the future design of highly conductive polymer electrolytes.

## Introduction

Poly(ionic liquid) (PIL) block copolymers have shown promise as solid polymer electrolytes (SPEs) in electrochemical systems, such as lithium-ion batteries^1−3^ and fuel cells.^4−8^ PILs exhibit excellent physicochemical properties, including high ionic conductivity,^9,10^ high chemical stability,^11−13^ and high electrochemical stability,^14^ which are highly desired properties for SPEs.^15,16^ When copolymerized with a mechanical support block, PIL block copolymers incorporate the electrochemical properties of the PIL block with the desired mechanical properties of the nonionic block through the self-assembling nanophase separated structure (i.e., morphology), resulting in robust SPEs.^17^

Various studies^18−25^ have demonstrated that the ionic conductivity of block copolymer electrolytes is highly dependent on the nanostructured morphology, such as in neutral block copolymers doped with salts or ionic liquids (ILs), single-ion conducting block copolymers, etc. The effect of morphology on conductivity can be quantified by the morphology factor (*f*), following the earlier work of gas permeability in AB diblock copolymers by Sax and Ottino,^26^ where the permeability or conductivity of the material is normalized by the conductivity of the conducting phase (or conductivity of the homopolymer) weighted by its volume fraction.^27−30^ Under circumstances where no tortuous paths, grain boundaries, or dead ends exist in the polymers, an ideal morphology factor varies for different morphology types, e.g., 1/3 for hexagonally packed one-dimensional (1D) cylinders, 2/3 for two-dimensional (2D) lamellae, and 1 for 3D gyroid networks.^31^ A morphology factor greater than 1 indicates that the morphology enhances conductivity over its pure homopolymer (with conducting volume fraction of 1) despite the reduction in conducting volume fraction, i.e., continuous nanodomains may accelerate ion conductivity.

Balsara and co-workers^21,22,30,32^ have extensively investigated the morphology and ion transport of polystyrene-*b*-poly(ethylene oxide) (SEO) diblock copolymer/lithium salt polyelectrolytes. In a hybrid block copolymer electrolyte (SEO/lithium salt), the ion conductivity of the polyelectrolyte increased with the occurrence of a lamellar-to-bicontinuous phase transition, and a high morphology factor close to 1 was observed for the bicontinuous phase.^22^ Park and co-workers^18^ investigated the conductivities of sulfonated block copolymer/IL polyelectrolytes with different self-assembled morphologies driven by different IL types. They observed that the gyroid morphology achieved a higher morphology factor (0.6–0.7) compared to lamellar and/or hexagonal cylinder morphologies (ca. 0.4). Another study by Park and co-workers^33^ also showed that the A15 lattice morphology obtained higher morphology factors (0.83–0.96) than the hexagonally packed cylinder morphology (0.42–0.69) in a phosphonated block copolymer/IL system. Choi et al.^19^ synthesized multiple compositions of a single-ion conducting AB diblock copolymer that exhibited cylindrical, lamellar, and network morphology types. The polymer having the network morphology exhibited a high ionic conductivity of 0.88 mS cm^–1^ at 150 °C, with a morphology factor of approximately 1, compared to morphology factors of 0.54–0.67 and <0.1 for the lamellar and cylindrical morphologies, respectively. The high conductivity and morphology factor can be attributed to an enhanced connectivity of conducting microdomains driven by the change to a network morphology. Hence, when designing conductive block copolymers, a nanostructured morphology with a 3D continuous domain for the conductive block is highly desirable.

**Figure 1:**
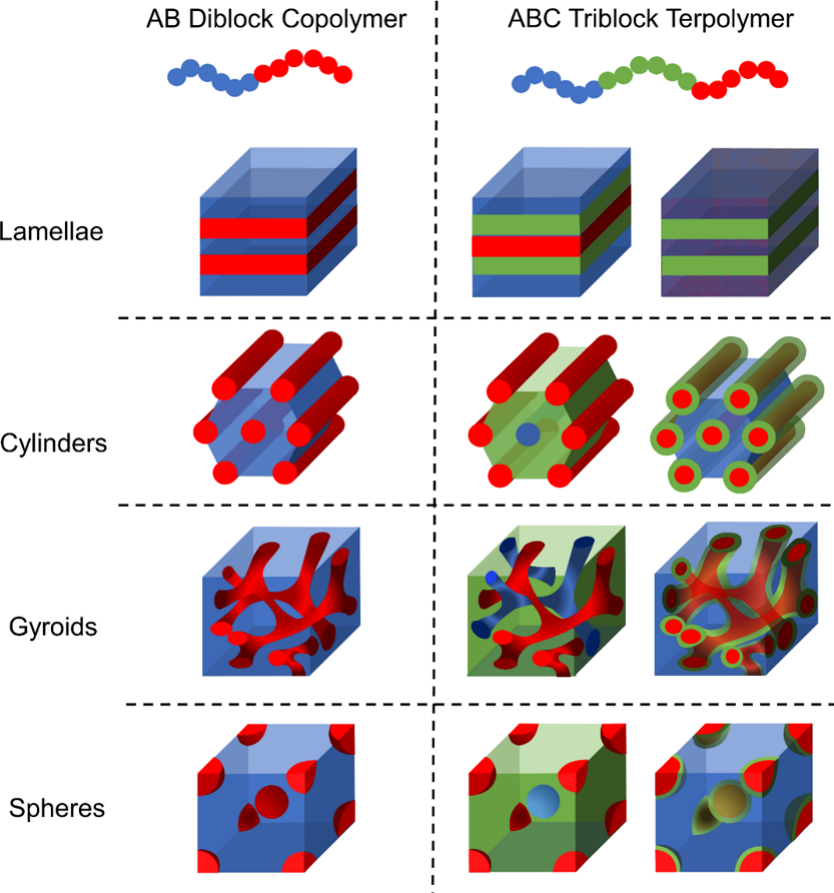
AB diblock copolymer architecture and morphologies (left) and ABC triblock terpolymer architecture and selected morphologies (right).

Figure 1 (left) illustrates the morphologies for AB diblock copolymers. Diblock copolymers only exhibit lamellae, hexagonally packed cylinders, Q^230^ gyroid (*Ia*3̅*d*), and spheres on a body-centered cubic (BCC) lattice^34^ of which the gyroid is the only 3D bicontinuous network. Additionally, the network morphology in diblock copolymers only occurs over a narrow composition range,^35−37^ limiting the ability to create diblock copolymers with 3D co-continuous domains. When compared to AB diblock copolymers, ABC triblock terpolymers offer access to more degrees of freedom in their design, including the block sequence (ABC vs ACB vs CAB), two unique compositions (ϕ_A_ and ϕ_B_), and three Flory–Huggins segmental interaction parameters (χ_AB_, χ_AC_, and χ_BC_), which can impact the morphology of the polymers.^38^ The rich phase behavior of ABC triblock terpolymers produces not only three-phase analogues of morphologies observed in AB diblock copolymers^39^ but also three-phase core–shell morphologies,^40^ co-continuous morphologies,^41,42^ and a wide range of exotic morphologies,^43^ illustrated in Figure 1 (right).

Studies on ABC triblock terpolymers were recently reviewed by Chang and Bates^39^ through the lens of the model proposed by Zheng and Wang in 1995.^38^ Zheng and Wang proposed that morphological behavior could be predicted from a set of six conditions based on two ratios of surface tensions, γ_1_ = σ_BC_/σ_AB_ and γ_2_ = σ_AC_/σ_AB_, where σ_XY_ is the surface tension between the X block and the Y block. Surface tension scales as the Flory–Huggins segmental interaction parameter, χ_XY_. Bailey and others^44^ simplified this into three categories. The first category, χ_AC_ ≥ χ_BC_ ≥ χ_AB_, results in a material where the end-blocks are more miscible with the midblock than they are with each other.^39,43^ This drives the end-blocks to microphase separate more strongly from each other than the midblock from either end-block. This situation is termed “unfrustrated”, and results in morphologies that are simple analogues of AB diblock morphologies, such as a three-phase lamellar morphology (L_3_)^45−47^ or spheres of A and C in a matrix of B (space group 221, *Pm*3*m*, also called the CsCl structure),^47^ although more complex structures are also observed, including the Q^214^ alternating gyroid^41,48^ and the penta-continuous core–shell Q^230^ gyroid.^41,42^ Both the second category (χ_BC_ ≥ χ_AC_ ≥ χ_AB_) and the third category (χ_BC_ ≥ χ_AB_ ≥ χ_AC_) are termed “frustrated” because the midblock is less miscible with at least one end-block than the end-blocks with each other.^39,43^ The third category is the “more frustrated” of the two, from this perspective. It is in these frustrated categories where the truly quixotic morphologies have been observed, in addition to the morphologies described above, particularly when the volume fraction of the midblock is low. Famous examples include the knitting pattern,^49^ spheres-on-spheres,^50^ cylinders-on-lamellae,^51^ cylinders-in-lamellae,^52^ and rings-on-cylinders.^53^

For instance, Epps et al.^41^ characterized 43 compositions of the linear poly(isoprene-*b*-styrene-*b*-ethylene oxide) (ISO) triblock terpolymers (unfrustated, χ_AB_ ≈ χ_BC_ < χ_AC_) and their morphology types. In addition to two- and three-phase lamellae, three network morphologies (the Q^230^ core–shell double gyroid phase, the O^70^ orthorhombic network phase, and the Q^214^ alternating gyroid phase) were found over an approximate composition window of 0.3 < ϕ_s_ < 0.6, 0.2 < ϕ_I_ < 0.5, and 0.1 < ϕ_O_ < 0.3. Chang and co-workers^54^ investigated the phase behavior of poly(styrene-*b*-isoprene-*b*-methyl methacrylate) (frustrated, χ_AC_ < χ_AB_ < χ_BC_) formed by solvent vapor annealing. Multiple exotic morphology types including cylinder-on-lamellar, core–shell cylinder, and cylinder-on-cylinder morphology were observed at different polymer compositions. Compared to diblock copolymers, the larger composition window for 3D continuous morphologies in ABC triblock terpolymers allows for a larger synthesis target and the ability to further fine-tune the compositions of the other blocks to achieve diverse exotic morphologies and desired polymer properties.

Several studies on ABC triblock terpolymers doped with lithium salts or ILs show that the presence of ionic species has a large effect on the morphology type. Epps et al.^55,56^ reported that the addition of salt significantly changes the phase behavior of poly(styrene-*b*-isoprene-*b*-ethylene oxide) (SIO) triblock terpolymers and leads to the disappearance of network morphologies, possibly due to the increasing effective χ parameters. Lodge and co-workers^57^ investigated the impact of IL doping on the morphology of poly[isoprene-*b*-(styrene-*co*-norbornenylethylstyrene)-*b*-ethylene oxide] (INSO) triblock terpolymer. The morphology changes from highly ordered O^70^ network morphology to hexagonally packed cylinders for the INSO/IL blends, especially at higher IL concentrations, attributed to interfacial energy change between blocks with the addition of ILs. The conductivities of the INSO/IL blends were not measurable, possibly due to isolated or nonpercolating conducting domains.

To date, the effect of the ion-conducting block composition on the morphology of single-ion conducting ABC triblock terpolymers (e.g., PIL ABC triblock terpolymer) is yet to be explored. To the best of the authors’ knowledge, the only study of a single-ion conducting ABC triblock terpolymer was conducted by Mayes and co-workers,^58^ where the authors investigated the impact of counterion placement (i.e., outside or inside the ion-conducting block) on ion conductivity. A phase diagram of single-ion conducting ABC triblock terpolymers has not been established, and a systematic study on the relationship between block composition, morphology, and ion conductivity has yet to be performed.

In this work, we construct a morphology phase diagram for PIL ABC triblock terpolymers with the intent of systematically exploring morphology type, identifying a composition window for each morphology, and measuring morphology factors. To achieve this, we synthesized 17 unique compositions of the PIL triblock terpolymer poly(S-*b*-VBMIm-TFSI-*b*-HA), where S is styrene, VBMIm-TFSI is vinylbenzyl methylimidazolium bis(trifluoromethanesulfonyl)imide, and HA is hexyl acrylate. Synthesis was accomplished via reversible addition–fragmentation chain transfer (RAFT) polymerization and postpolymerization modifications, such as functionalization and ion exchange reactions. Based on this ABC chemistry with an immiscible PIL midblock, by definition, these polymers fall in the highly frustrated category (χ_BC_ ≥ χ_AB_ ≥ χ_AC_) described above, as explained in more detail in the Results and Discussion sections of this manuscript. Each composition of this polymer was characterized with ^1^H nuclear magnetic resonance (NMR) spectroscopy, size exclusion chromatography (SEC), elemental analysis (EA) and attenuated total reflectance Fourier transform infrared spectroscopy (ATR-FTIR) to confirm chemical composition and structure, differential scanning calorimetry (DSC) to determine the glass transition temperatures, and small-angle X-ray scattering (SAXS) to investigate the morphology types as a function of polymer composition. High-angle annular dark field scanning transmission electron microscopy (HAADF-STEM) was performed on select triblock terpolymers to verify the morphology types determined by SAXS. Additionally, ionic conductivity at various temperatures was measured with electrochemical impedance spectroscopy (EIS) and normalized to determine the morphology factors. This study reveals the impact of morphology type on the ion conductivity in a single-ion conducting ABC triblock terpolymer for the first time and provides a guide for further synthesis of highly conductive PIL ABC triblock terpolymer SPEs.

## Experimental Methods

### Materials

2-Cyanobutanyl-2-yl 3,5-dimethyl-1H-pyrazole-1-carbodithioate (chain transfer agent (CTA), ≥95%, Boron Molecular), toluene (anhydrous, 99.8%, Sigma-Aldrich), tetrahydrofuran (THF, anhydrous, ≥99.9%, inhibitor-free, Sigma-Aldrich), tetrahydrofuran (HPLC THF, inhibitor-free, for HPLC, ≥99.9%, Sigma-Aldrich), 1-methylimidazole (ReagentPlus, 99%, Sigma-Aldrich), chloroform-d (CDCl_3_, 99.96 atom % D, contains 0.03% (v/v) TMS, Sigma-Aldrich), dimethyl sulfoxide-*d*_6_ (DMSO-*d*_6_, 99.9 atom % D, contains 0.03% (v/v) TMS, Sigma-Aldrich), *N*, *N*-dimethylformamide (DMF, anhydrous, 99.8%, Sigma-Aldrich), hexane (anhydrous, 95%, Sigma-Aldrich), acetone (ACS reagent, ≥99.5%, Sigma-Aldrich), and methanol (ACS reagent, ≥99.8%, Sigma-Aldrich) were used as received. 1,1′-Azobis(cyclohexanecarbonitrile) (ACHN, 98%, Sigma-Aldrich) was purified via recrystallization twice from methanol. Bis(trifluoromethane)sulfonimide lithium salt (LiTFSI, 99.95% trace metals basis, Sigma-Aldrich) was dried at 110 °C under dynamic vacuum for 24 h before use. Styrene (S, ≥99%, contains 4-*tert*-butylcatechol as stabilizer, Sigma-Aldrich), vinylbenzyl chloride (VBC, mixture of 3- and 4-isomers, 97%, contains 700–1100 ppm nitromethane as inhibitor, 50–100 ppm *tert*-butylcatechol as inhibitor, Sigma-Aldrich), and hexyl acrylate (HA, 98%, contains 100 ppm hydroquinone as inhibitor, Sigma-Aldrich) were purified by passing dropwise through a hollow glass tube packed with inhibitor removers for their respective inhibitors (Sigma-Aldrich). The argon-filled glovebox (mBraun) was maintained at both water and oxygen concentrations <5 ppm and environmental pressure between 1 and 8 mbar. CR2032 coin cell cases with O-rings (diameter: 20 mm, thickness: 3.2 mm, MTI Corporation), stainless steel spacers (diameter: 15.5 mm, thickness: 1.0, 0.5, and 0.2 mm, MTI Corporation), and stainless steel wave springs (height: 1.2 mm, thickness: 0.3 mm, MTI Corporation) were used as received for ion conductivity measurements. Mylar PET release liner substrates (Grade 26965, 0.0762 mm, LOPAREX) were used as received. Deionized water (DI H_2_O, resistivity ca. 16 MΩ) was used as appropriate.

### Reflux Reaction Procedure

**Scheme 1:**
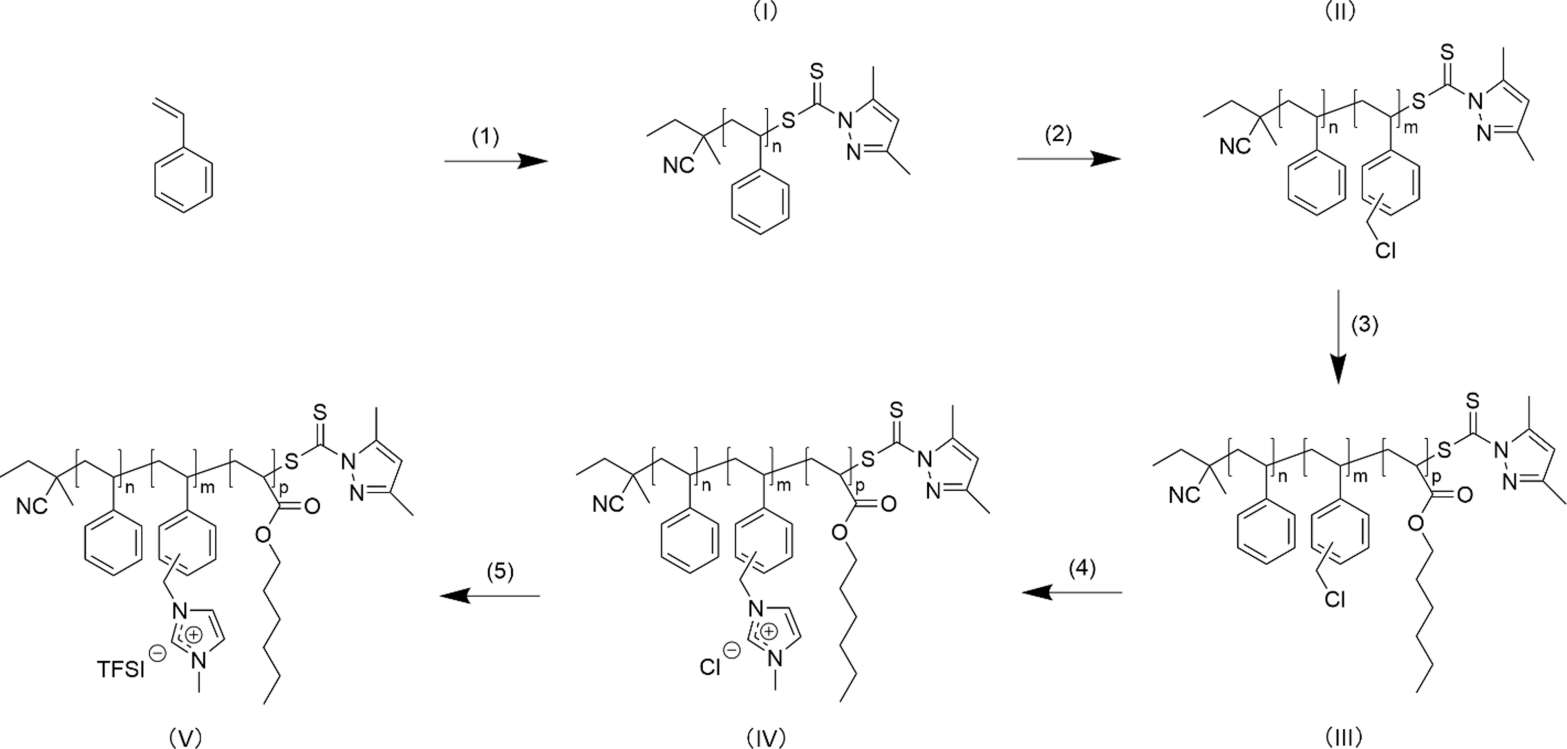
Synthesis of PIL ABC Triblock Terpolymer Poly(S-*b*-VBMIm-TFSI-*b*-HA) (1) 2-cyanobutanyl-2-yl 3,5-dimethyl-1H-pyrazole-1-carbodithioate (CTA), toluene, reflux, 20 h; (2) VBC, ACHN, THF, reflux; (3) HA, ACHN, THF, reflux; (4) 1-methylimidazole, DMF, 80 °C, 48 h; (5) LiTFSI, DMF, 50 °C, 48 h. Reaction times for (2) and (3) listed in Table S2, Supporting Information.

All polymerization reactions (Scheme 1; products I, II, and III) in this work were performed following a reflux reaction procedure as described in detail previously.^59^ A summary of polymer structure and all RAFT polymerization reaction conditions are listed in Tables S1 and S2, respectively. In brief, monomer and precursor (CTA or polymer reactant) were mixed with solvent in a three-neck round-bottom-flask. The amount of solvent was equal to the amount of monomer by weight. The central neck of the flask was connected to a reflux condenser, which was further connected to a Schlenk line with nitrogen purge and bubbler. The other two necks were sealed with rubber septa. The reacting mixture was degassed with nitrogen, after which the reactor was placed in an oil bath and heated to reflux temperature. While the reactor was heating, initiator was dissolved in a solvent in a separate vial (for specified reactions described below), then degassed for 5 min with nitrogen. The initiator solution was injected into the reacting mixture immediately after observing reflux to start the reaction. After reacting for the specified time, the reaction was terminated by precipitating dropwise into methanol. The resulting polymer was twice precipitated dropwise in methanol, filtered, and dried under a dynamic vacuum in an oven at room temperature for 24 h.

### Synthesis of PS Macro-CTA

**Figure 2:**
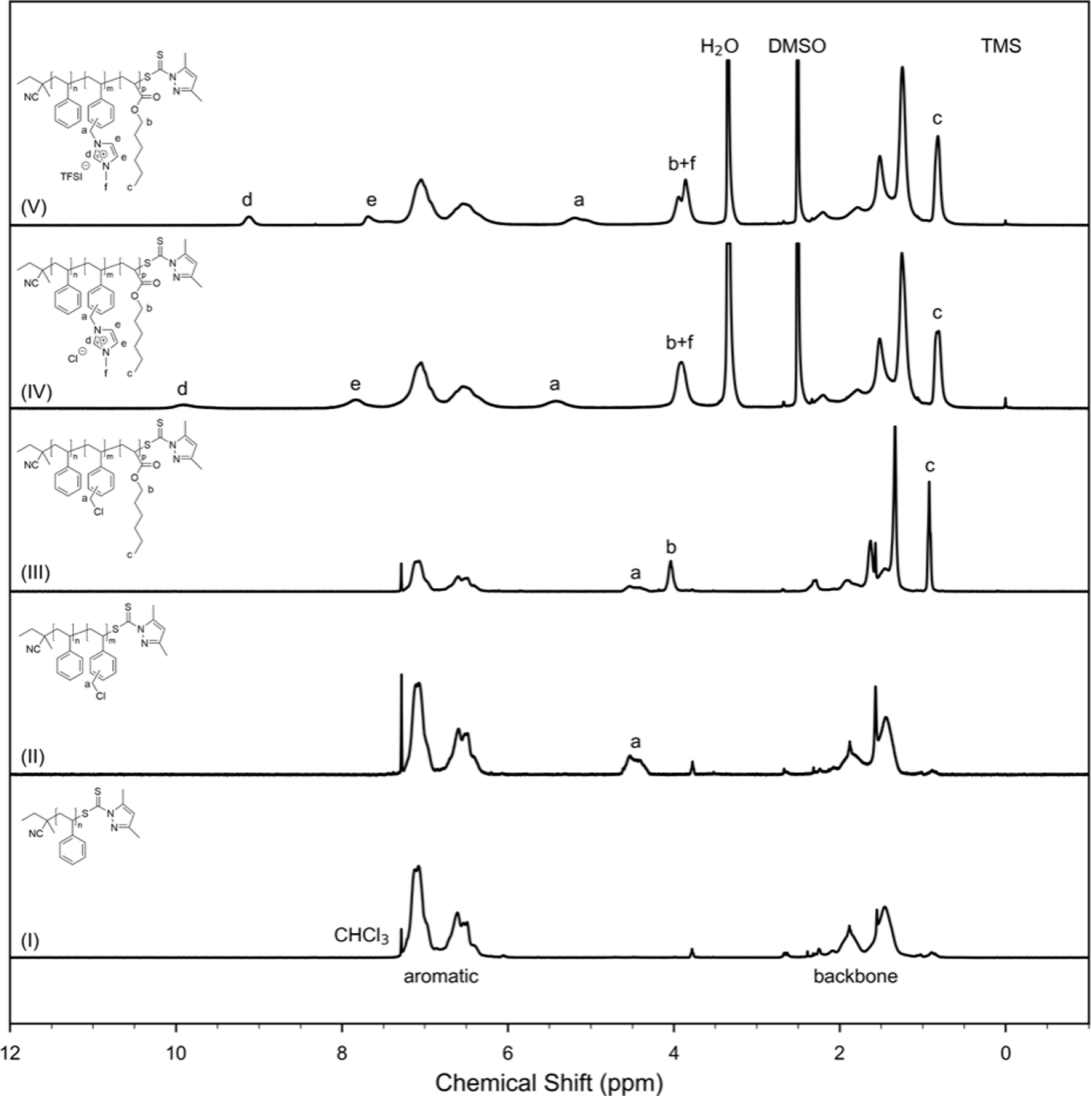
Representative ^1^H NMR spectra and peak assignments of (I) PS macro-CTA, (II) poly(S-*b*-VBC), (III) poly(S-*b*-VBC-*b*-HA), (IV) poly(S-*b*-VBMIm-Cl-*b*-HA), and (V) poly(S-*b*-VBMIm-TFSI-*b*-HA). H_2_O and DMSO solvent peaks are not shown in entirety to improve figure clarity. ^1^H NMR spectra for all 17 polymers included in this study can be found in Figure S1.

The synthesis of poly(styrene) (PS) macro-CTA is shown in Scheme 1(1), following the reflux reaction procedure described above with the following: monomer: styrene (S); solvent: toluene; precursor: CTA; initiator: none. The resulting polymer was twice precipitated dropwise in methanol, filtered, dried under dynamic vacuum in an oven at room temperature for 24 h, and then stored in sealed glass containers at −15 °C. Reaction details are listed in Table S2. Product details are listed in Table S3. ^1^H NMR (500 MHz, CDCl_3_, 23 °C, Figure 2(I)) δ (ppm): 7.22–6.28 (m, 5H, C_6_*H*_5_), 2.40–1.66 (m, 1H, CH_2_C*H*), 1.66–1.12 (m, 2H, C*H*_2_CH).

### Synthesis of Poly(S-*b*-VBC)

The synthesis of poly(S-*b*-VBC) is shown in Scheme 1(2), following the reflux reaction procedure described above with the following: monomer: VBC; solvent: THF; precursor: PS macro-CTA; initiator: ACHN. The resulting polymer was twice precipitated dropwise in methanol, filtered, dried under dynamic vacuum in an oven at room temperature for 24 h, and then stored in sealed glass containers at −15 °C. Reaction details are listed in Table S2. Product details are listed in Table S3. ^1^H NMR (400 MHz, CDCl_3_, 23 °C, Figure 2(II)) δ (ppm): 7.22–6.28 (m, 9H, C_6_*H*_5_ and C_6_*H*_4_), 4.66–4.08 (m, 2H, C*H*_2_Cl), 2.40–1.66 (m, 1H, CH_2_C*H*), and 1.66–1.12 (m, 2H, C*H*_2_CH).

### Synthesis of Poly(S-*b*-VBC-*b*-HA)

The synthesis of poly(S-*b*-VBC-*b*-HA) is shown in Scheme 1(3), following the reflux reaction procedure described above with the following: monomer: HA; solvent: THF; precursor: poly(S-*b*-VBC); initiator: ACHN. The resulting polymer was twice precipitated dropwise in methanol, filtered, dried under dynamic vacuum in an oven at room temperature for 24 h, and then stored in sealed glass containers at room temperature. Reaction details are listed in Table S2. Product details are listed in Table S3. ^1^H NMR (400 MHz, CDCl_3_, 23 °C, Figure 2(III)) δ (ppm): 7.22–6.28 (m, 9H, C_6_*H*_5_ and C_6_*H*_4_), 4.66–4.08 (m, 2H, C*H*_2_Cl), 4.04–3.76 (m, 2H, COOC*H*_2_), 2.40–1.66 (m, 1H, CH_2_C*H*), 1.66–1.12 (m, 2H, C*H*_2_CH).

### Synthesis of Poly(S-*b*-VBMIm-Cl-*b*-HA)

The synthesis of poly(S-*b*-VBMIm-Cl-*b*-HA) is shown in Scheme 1(4). Poly(S-*b*-VBC-*b*-HA) and 1-methylimidazole (five times molar excess relative to the number of repeat units in the VBC block) were dissolved into DMF (poly(S-*b*-VBC-*b*-HA)/DMF (1/4) (w/w)) in a 125 mL flask, which was subsequently sealed with a rubber septum. The sealed flask was then placed into an oil bath at 80 °C and stirred for 48 h. The resulting polymer was precipitated into hexane, then washed 10 times in hexane by decanting and replacing the hexane every 6 h, and then washed either in a DI water/methanol mixture (1/1 v/v) or acetone (based on solubility) 10 times by decanting and replacing the washing fluid every 3 h to remove the excess DMF and 1-methylimidazole, filtered, then dried under dynamic vacuum in an oven at room temperature for 24 h. Product details are listed in Table S3 and EA results are listed in Table S4. ^1^H NMR (400 MHz, DMSO-*d*_6_, 23 °C, Figure 2(IV)) δ (ppm): 10.37–9.46 (s, 1H, NC*H*N), 8.28–7.58 (m, 2H, NC*H*C*H*N), 7.58–5.95 (m, 9H, C_6_*H*_5_ and C_6_*H*_4_), 5.82–4.97 (m, 2H, C*H*_2_N), 4.19–3.56 (m, 2H, COOC*H*_2_), 2.42–1.71 (m, 1H, CH_2_C*H*), 1.71–0.61 (m, 2H, C*H*_2_CH).

### Synthesis of Poly(S-*b*-VBMIm-TFSI-*b*-HA)

The synthesis of poly(S-*b*-VBMIm-TFSI-*b*-HA) is shown in Scheme 1(5). Poly(S-*b*-VBMIm-Cl-*b*-HA) and LiTFSI (five times molar excess relative to the number of repeat units in the VBMIm-Cl block) were dissolved in DMF (poly(S-*b*-VBMIm-Cl-*b*-HA)/DMF (1/4) (w/w)) in a 125-mL flask which was subsequently sealed with a rubber septum. The sealed flask was then placed into an oil bath at 50 °C and stirred for 48 h. The resulting polymer was precipitated once into a DI water/methanol mixture (50/50 (v/v)) then washed 10 times with DI water by decanting and replacing the DI water every 3 h to remove excess DMF and LiTFSI, filtered, and dried under dynamic vacuum in an oven at room temperature for 24 h. Product details are listed in Table S3 and EA results are listed in Table S5. ^1^H NMR (400 MHz, DMSO-*d*_6_, 23 °C, Figure 2(V)) δ (ppm): 9.32–8.96 (s, 1H, NC*H*N), 7.77–7.31 (m, 2H, NC*H*C*H*N), 7.31–5.90 (m, 9H, C_6_*H*_5_ and C_6_*H*_4_), 5.41–4.72 (m, 2H, C*H*_2_N), 4.33–3.51 (m, 2H, COOC*H*_2_), 2.23–1.71 (m, 1H, CH_2_C*H*), 1.71–0.61 (m, 2H, C*H*_2_CH).

### Solution Casting Polymer Films

Polymer film samples were prepared for SAXS analysis, transmission electron microscopy (TEM), and ion conductivity measurements via a solution casting method. Polymer was dissolved into THF (5/95) (w/w, polymer/THF) overnight to ensure complete dissolution. The solutions were then cast onto Teflon Petri dishes and covered under a THF-rich environment (2 × 50 mL THF covered by Pyrex dish), where the solvent was allowed to evaporate for 48 h. The films were then removed from THF environment to further evaporate solvent for 12 h at room temperature and then placed under dynamic vacuum at room temperature for 12 h. Subsequently, the films were annealed in a vacuum oven at 125 °C for 48 h under dynamic vacuum, and then transferred into an argon glovebox for storage. Samples for SAXS and TEM analysis were removed from the Teflon Petri dishes and transported in vials sealed under the glovebox environment. Samples for ion conductivity measurements were stored between two layers of silicon-coated Mylar PET films in the glovebox.

### Characterization

Chemical structure, purity, block composition ratio, degree of functionalization, and ion exchange of all polymers were confirmed by NMR spectroscopy. PS macro-CTA, poly(S-*b*-VBC), and poly(S-*b*-VBC-*b*-HA) were characterized by ^1^H NMR spectroscopy using a Bruker Avance Neo 400 MHz spectrometer at 25 °C with CDCl_3_ as the solvent. The chemical shifts were referenced to chloroform at 7.27 ppm. Poly(S-*b*-VBMIm-Cl-*b*-HA) was characterized by ^1^H NMR spectroscopy at 25 °C with DMSO-*d*_6_ as the solvent. The chemical shifts were referenced to tetramethylsilane (TMS) at 0.00 ppm. Poly(S-*b*-VBMIm-TFSI-*b*-HA) was characterized by ^1^H and ^19^F NMR spectroscopy at 25 °C with DMSO-*d*_6_ as the solvent. The chemical shifts were referenced to TMS at 0.00 ppm. Degree of functionalization and ion exchange were further confirmed using EA. EA was performed by Atlantic Microlab, Inc., Norcross, GA.

Chemical structure was further characterized by ATR-FTIR spectroscopy. Infrared spectroscopy was performed at room temperature with a Fourier transform infrared spectrometer (Nicolet 6700 series; Thermo Electron Corporation) using a single reflection diamond attenuated total reflectance (ATR) accessory (Specac; Quest). All infrared spectra were collected using a liquid-nitrogen-cooled mercury–cadmium-telluride (MCT) detector at 32 scans per spectrum with a resolution of 4 and data spacing 1.928 cm^–1^. The spectra were corrected with a background subtraction of the spectrum of the bare ATR crystal.

The molecular weights and molecular weight distributions of all PS macro-CTA, poly(S-*b*-VBC), and poly(S-*b*-VBC-*b*-HA) polymers were determined by SEC using a Waters GPC system equipped with a THF Styragel column (Styragel@HR 5E, effective separation of molecular weight range: 2–4000 kg mol^–1^) and a 2414 reflective index (RI) detector. All measurements were performed at 40 °C, where THF was used as the mobile phase at a flow rate of 1.0 mL min^–1^. PS standards (Shodex, Japan) with molecular weights ranging from 2.97 to 983 kg mol^–1^ were used for calibration.

Glass transition temperatures (*T*_g_s) were determined by DSC analysis (Q200, TA Instruments). Experiments were performed using a heat/cool/heat method over a temperature range of −140 to 200 °C at a heating rate of 10 °C min^–1^ under a nitrogen environment. *T*_g_s were determined by the midpoint method on the second heating cycle. Degradation temperatures (*T*_d_s) were determined by thermogravimetric analysis (Q50, TA Instruments). Polymer samples were heated from ambient temperature to 900 °C at a rate of 10 °C min^–1^ in nitrogen at a flow rate of 60 mL min^–1^. *T*_d_s were determined as the temperature at which 95% of the starting sample mass remained (see results in Table S3).

The bulk morphology of poly(S-*b*-VBMIm-TFSI-*b*-HA) polymers at all compositions synthesized was characterized by SAXS. SAXS data were collected using a Xenocs “Xeuss 3.0 HR” instrument with 8.04 keV photons generated by a Rigaku 007HF rotating anode X-ray generator. The photons were collimated using a focusing optic and two scatterless slit apertures, producing a well-aligned incident beam with wavelength (λ) of 1.5418 Å. Data were collected using a Dectris PilatusR 300k solid-state detector at two sample-to-detector distances, 1800 and 900 mm, to give a combined angular range of 0.003 Å^–1^ < *q* < 0.3 Å^–1^, where *q* is the modulus of the scattering vector, such that *q* = 4π sin(θ)/λ for a scattering angle of 2θ. Two-dimensional data were azimuthally averaged to generate one-dimensional data, *I*(*q*), for analysis. The instrument configuration was calibrated using silver behenate, and data were placed on an absolute scale through normalization by transmitted flux. Data processing and analysis were performed using Wavemetrics Igor Pro v8 and procedures available for download from Argonne National Laboratory.^60,61^

Additional morphological analysis using high-angle annular dark field (HAADF) scanning transmission electron microscopy (STEM) was performed on selected samples. Ultrathin specimens approximately 60 nm thick were prepared by ultramicrotomy. Sections were cut using a Leica UC7 either at room temperature or cooled to approximately 10 °C below sample *T*_g_ using a Leica FC7 cryogenic cooling attachment. In most cases, sections were collected on TEM grids prepared with lacey carbon support films. HAADF-STEM was performed on the resulting sections using a JEOL JEM-2100F field-emission instrument operated at 200 kV accelerating voltage. HAADF-STEM often allows polymers to be imaged directly with no staining or other contrast enhancement applied due to the high contrast and small probe size (nominally 0.2 nm). The HAADF-STEM camera length was set to 10.6 cm, corresponding to an angle range of 24.1–54.3 mrad. Images were collected using Gatan Digital Micrograph 3. Mitchel’s Digital Micrograph script was used to calculate the mean free paths for single-component microphase separated domains.^62^

The ionic conductivities of poly(S-*b*-VBMIm-TFSI-*b*-HA) polymers were measured using EIS (Solartron, 1260 impedance analyzer, 1287 electrochemical interface, Zplot software) over a frequency range of 0.5–10^6^ Hz with an AC amplitude of 10 mV. Through-plane conductivity measurements were carried out in a two-electrode coin cell apparatus. Polymer films were punched into circular films (16 mm in diameter) and sandwiched between 2 to 4 stainless steel spacers (thickness: 1.0, 0.5, and 0.2 mm; diameter: 15.5 mm) in a CR2032 coin cell. The coin cells were pressed twice using an electric coin cell crimping machine (MTI Corporation, MSK-160D) in a glovebox at room temperature to ensure a proper seal. The real impedance (*R*, resistance) was determined from the equivalent circuit regression of the Nyquist plot. Temperature-dependent conductivities at a temperature range of 30–100 °C were collected in an environmental chamber (Maccor, MTC-020). Samples were exposed to the corresponding temperature for 1 h to reach equilibrium conditions prior to the conductivity measurements. Conductivity was calculated by using the following equation:
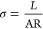
1where *L* is the polymer film thickness and *A* is the cross-sectional area of the film (*A* = π*d*^*2*^/4, *d* is the diameter of the blocking electrodes). Six conductivity measurements were performed at each equilibrium condition and the values reported are an average of these steady-state measurements.

## Results and Discussion

### PIL ABC Triblock Terpolymer Synthesis

Seventeen unique compositions of a PIL ABC triblock terpolymer, poly(S-*b*-VBMIm-TFSI-*b*-HA), were synthesized via RAFT polymerization, followed by quaternization and anion exchange reactions (Scheme 1). Sequential RAFT polymerizations of styrene (S), a PIL precursor, VBC, and HA yielded a neutral triblock terpolymer. Polymer composition was varied by modifying reaction conditions and reaction time (listed in Table S2). The VBC block was then quaternized with 1-methylimidazole, and subsequently converted into the final ABC PIL triblock terpolymer via anion exchange reactions in the presence of bis(trifluoromethane)sulfonimide lithium salt (LiTFSI).

**Figure 3:**
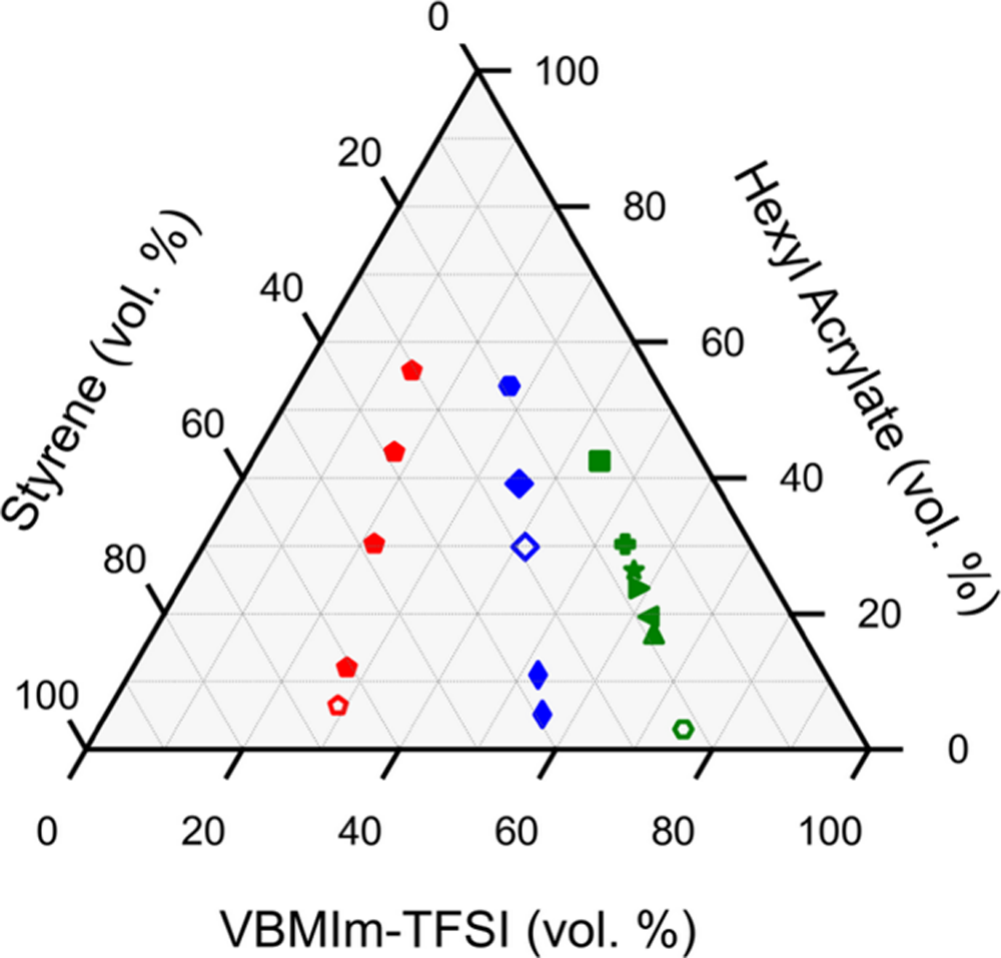
Compositions of synthesized PIL ABC triblock terpolymer, poly(S-*b*-VBMIm-TFSI-*b*-HA), with 6 (red), 19 (blue), and 45 (green) repeat units of VBMIm-TFSI (or PIL).

The chemical structure and purity of all 17 triblock terpolymers were confirmed by ^1^H NMR spectroscopy. The spectra for one representative composition of the polymer at all stages of the synthesis (as shown in Scheme 1) are shown in Figure 2. Specific peak assignments are listed in the corresponding synthesis section for each polymer. The degree of polymerization of the S block was determined by SEC and confirmed with ^1^H NMR spectroscopy by the integration ratio of the protons on the aromatic carbons to the peak at 3.76 ppm (i.e., the CCHC proton on the end group). Chain extension of VBC on PS macro-CTA is confirmed by the appearance of peak a for the protons on the methylene group adjacent to the 3 or 4 position of the benzene ring (Figure 2(II)). Chain extension of HA on poly(S-*b*-VBC) is further evidenced by the appearance of peak b, for the protons on the alkyl carbon adjacent to the acrylate group on the HA block, and peak c, for the protons at the end of the alkyl carbons on the HA block (Figure 2(III)). The degree of polymerization of VBC and HA blocks was calculated by the integration ratio of the aromatic peaks to peak a and peak b, respectively. Successful functionalization of the VBC block is indicated by the appearance of peaks d, e, and f (Figure 2(IV)), where the integration ratio between peaks a, d, and e demonstrates that the poly(S-*b*-VBMIm-Cl-*b*-HA) is fully functionalized. Successful ion exchange reactions were confirmed by the presence of the TFSI anion from a ^19^F NMR peak at −78.7 ppm (Supporting Information, Figure S2). Infrared bands at 1348, 1328, 1180, 1133, and 1053 cm^–1^ (Supporting Information, Figure S3) are also indicative of SO_2_ asymmetric stretching,^63,64^ SO_2_ asymmetric stretching,^64^ CF_3_ stretching,^65,66^ SO_2_ symmetric stretching,^64^ and S–N–S antisymmetric stretching^67^ in the TFSI ion, respectively. EA results (Supporting Information, Tables S4 and S5) further confirm the success of quaternization and ion exchange reactions, where a close match between determined compositions and theoretical calculations was observed. The nomenclature, molecular weights, dispersities, and volume fractions of the 17 poly(S-*b*-VBMIm-TFSI-*b*-HA) polymers are listed in Table 1, with calculation details given in the Supporting Information. The sequential nature of the synthesis produced materials that have identical degree of polymerization of S (46 repeat units), three different degrees of polymerization for VBMIm-TFSI (6, 19, or 45 repeat units), and various HA compositions. This naturally leads to the grouping of polymers on the ternary phase diagram based on composition, as illustrated by symbol color in Figure 3 and Table 1. Note that, the colors of the symbol were selected based on the numbers of repeat units of VBMIm-TFSI (or PIL) block, (i.e., red for 6 repeat units, blue for 19 repeat units, and green for 45 repeat units of the PIL block). The symbols of the polymers were selected based on the morphology types determined from SAXS and TEM results, i.e., same symbols represent polymers with the same morphology types. The unique symbols of polymer samples of 46–45–27, 46–45–32, 46–45–41, 46–45–47, 46–45–57, and 46–45–97 were due to the necessity to represent the conductivity values for these polymers.

**Table 1:**
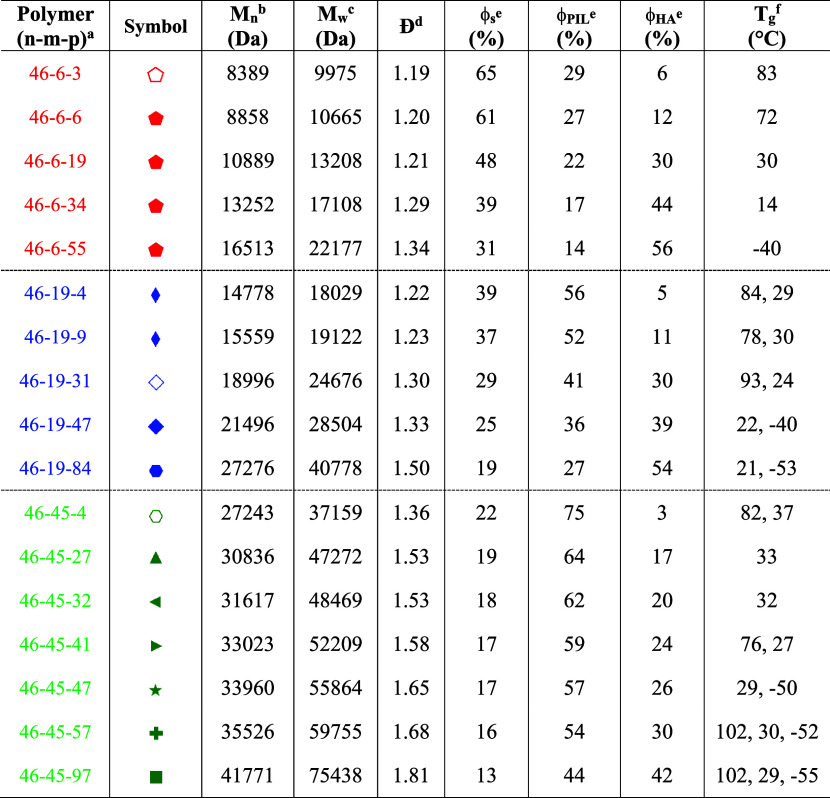
Nomenclature, Symbol, Molecular Weights, Dispersities (*Đ*), Volume Fractions, and Thermal Properties of Poly(S-*b*-VBMIm-TFSI-*b*-HA)

a Numbers (*n*, *m*, and *p*) represent the number of repeat units in the S, VBMIm-TFSI, and HA blocks, respectively, as determined by ^1^H NMR spectroscopy.

b Theoretical value calculated based on compositions determined by ^1^H NMR spectroscopy.

c Theoretical value calculated based on compositions determined by ^1^H NMR spectroscopy and dispersities determined by SEC.

d Determined by SEC of the neutral triblock terpolymer poly(S-*b*-VBC-*b*-HA).

e Volume fractions calculated from density of polystyrene (1.04 g cm^–3^), poly(VBMIm-TFSI) (1.405 g cm^–3^), and poly(hexyl acrylate) (1.05 g cm^–3^) (volume fraction calculation can be found in Supporting Information, Sections S2 and S3, Tables S6 and S7).

f Determined by DSC, multiple *T*_g_s separated by comma (,).

### Thermal Transitions

**Figure 4:**
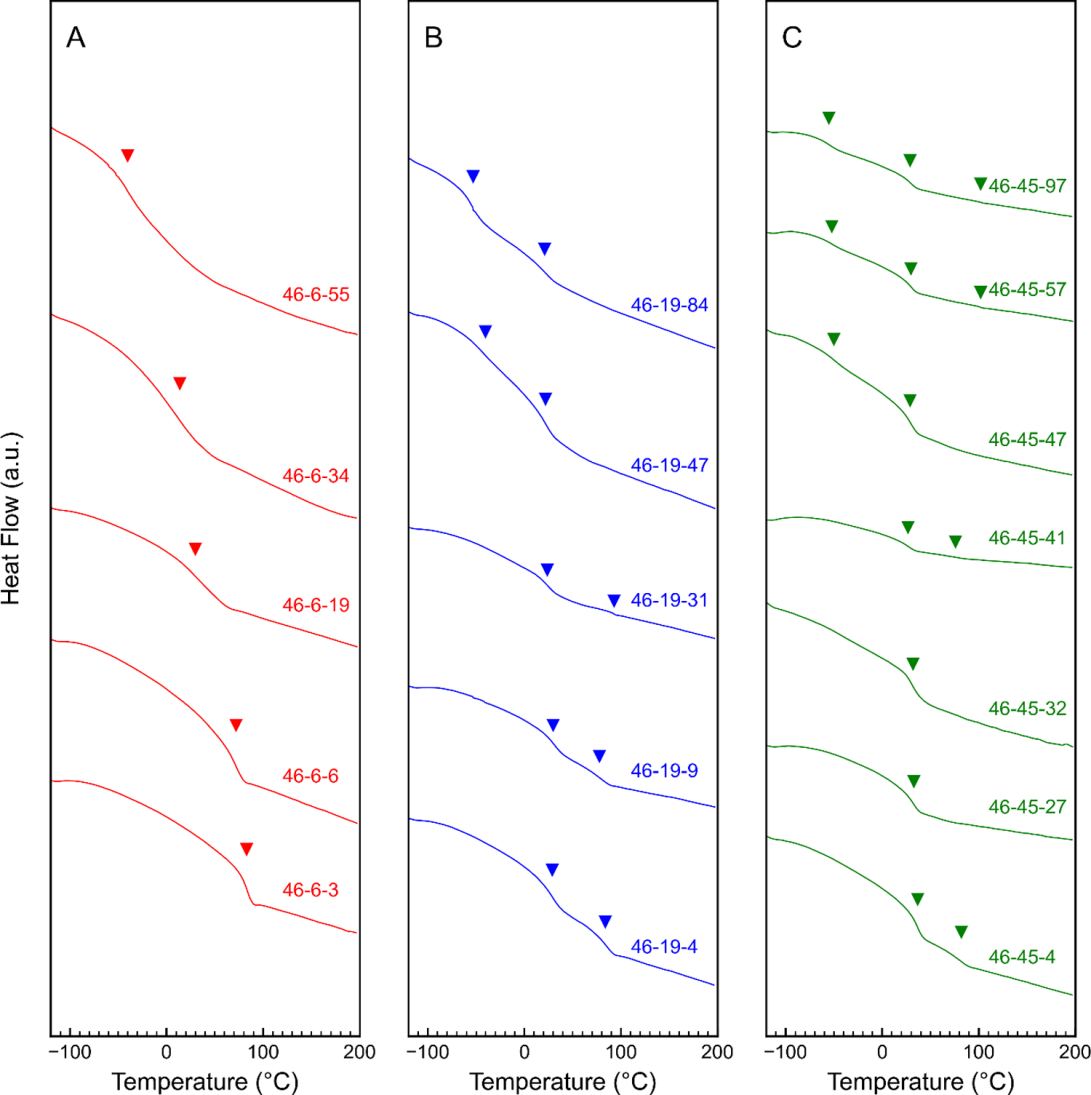
DSC profiles of poly(S-*b*-VBMIm-TFSI-*b*-HA) polymers with (A) 6 repeat units (red), (B) 19 repeat units (blue), and (C) 45 repeat units (green) of VBMIm-TFSI or PIL. Glass transition temperatures are indicated by inverted triangles; numerical values are listed in Table 1.

In general, the glass transition temperatures for all poly(S-*b*-VBMIm-TFSI-*b*-HA) occur at one or more of three temperatures: ca. 90 °C, ca. 30 °C, and ca. −50 °C (Figure 4 and Table 1). These temperatures correspond to the glass transition temperatures of the S, VBMIm-TFSI (or PIL), and HA blocks, respectively. The presence of multiple *T*_g_s in a profile may suggest microphase separation with each *T*_g_ representing one of the phase-separated domains. Figure 4A shows the DSC profiles for poly(S-*b*-VBMIm-TFSI-*b*-HA) containing six repeat units of the VBMIm-TFSI block (14% < ϕ_PIL_ < 29%). All five polymers show a single *T*_g_ ranging from 83 °C (46–6–3) to −40 °C (46–6–55). The presence of only a single *T*_g_ may be due to the low degree of polymerization of the VBMIm-TFSI block, which therefore may not be detected as a distinct *T*_g_. Additionally, the change in *T*_g_ with increasing HA block repeat units might suggest the absence of well-defined domains of S and HA, where separate *T*_g_s for S and HA block were not observed in this set of polymers. Figure 4B shows the DSC profiles for poly(S-*b*-VBMIm-TFSI-*b*-HA) containing 19 repeat units of the VBMIm-TFSI block (27% < ϕ_PIL_ < 56%). Each profile shows two *T*_g_s, suggesting a microphase-separated morphology for all five polymers. With higher VBMIm-TFSI block composition, all polymers show a *T*_g_ at ca. 30 °C corresponding to the VBMIm-TFSI block. Similar to the first group of polymers (6 repeat units of the PIL or VBMIm-TFSI block), a *T*_g_ at ca. – 50 °C (representing the HA block) appears as HA content increases, and the *T*_g_ at ca. 90 °C (representing the S block) disappears. Figure 4C shows the DSC profiles for poly(S-*b*-VBMIm-TFSI-*b*-HA) polymers containing 45 repeat units of the VBMIm-TFSI block (44% < ϕ_PIL_ < 75%). Each of these polymers again shows a *T*_g_ at ca. 30 °C representing the VBMIm-TFSI block, attributed to the high PIL block content. All high HA content polymers (ϕ_HA_ > 25%) show a distinct *T*_g_ at ca. −50 °C. At comparable content of VBMIm-TFSI or PIL (40% < ϕ_PIL_ < 55%) and HA blocks (30% < ϕ_HA_ < 45%), DSC profiles of 46–45–57 and 46–45–97 show three *T*_g_s, one at each ca. 90 °C, ca. 30 °C, and ca. – 50 °C, suggesting an ABC triblock terpolymer morphology with three distinct separated domains.

### Morphology

The morphology of poly(S-*b*-VBMIm-TFSI-*b*-HA) polymers was characterized with SAXS and HAADF-STEM. Morphology was assigned by comparing the observed Bragg diffraction peak positions to their expected positions,^41,68−70^ and compared to TEM images of the polymer samples. Expected diffraction peak locations for common morphologies for AB diblock copolymers (BCC spheres, hexagonally packed cylinders (C), lamellae (L), and gyroid (Q^230^)) and ABC triblock terpolymers (core–shell double gyroid (Q^230^), alternating gyroid (Q^214^), hexagonally packed core–shell cylinders (C_CS_), and the orthorhombic network (O^70^)) are listed in the Supporting Information (Table S8) for reference. In TEM, higher mass regions scatter more electrons from the incident electron beam while lower mass regions scatter fewer electrons. In dark field TEM, where scattered electrons are collected by the HAADF detector. The high-mass regions appear bright, and the low mass regions appear dark. To distinguish between microphase-separated domains of the different terpolymer components, the mean free path, which is a measure of average mass, was calculated for each component. The mean free paths for S, HA, and VBMIm-TFSI were calculated to be 146, 135, and 128 nm, respectively, indicating that domains comprising the PIL or VBMIm-TFSI block will be brightest in the HAADF-STEM images, while those comprising S will be darkest.

**Figure 5:**
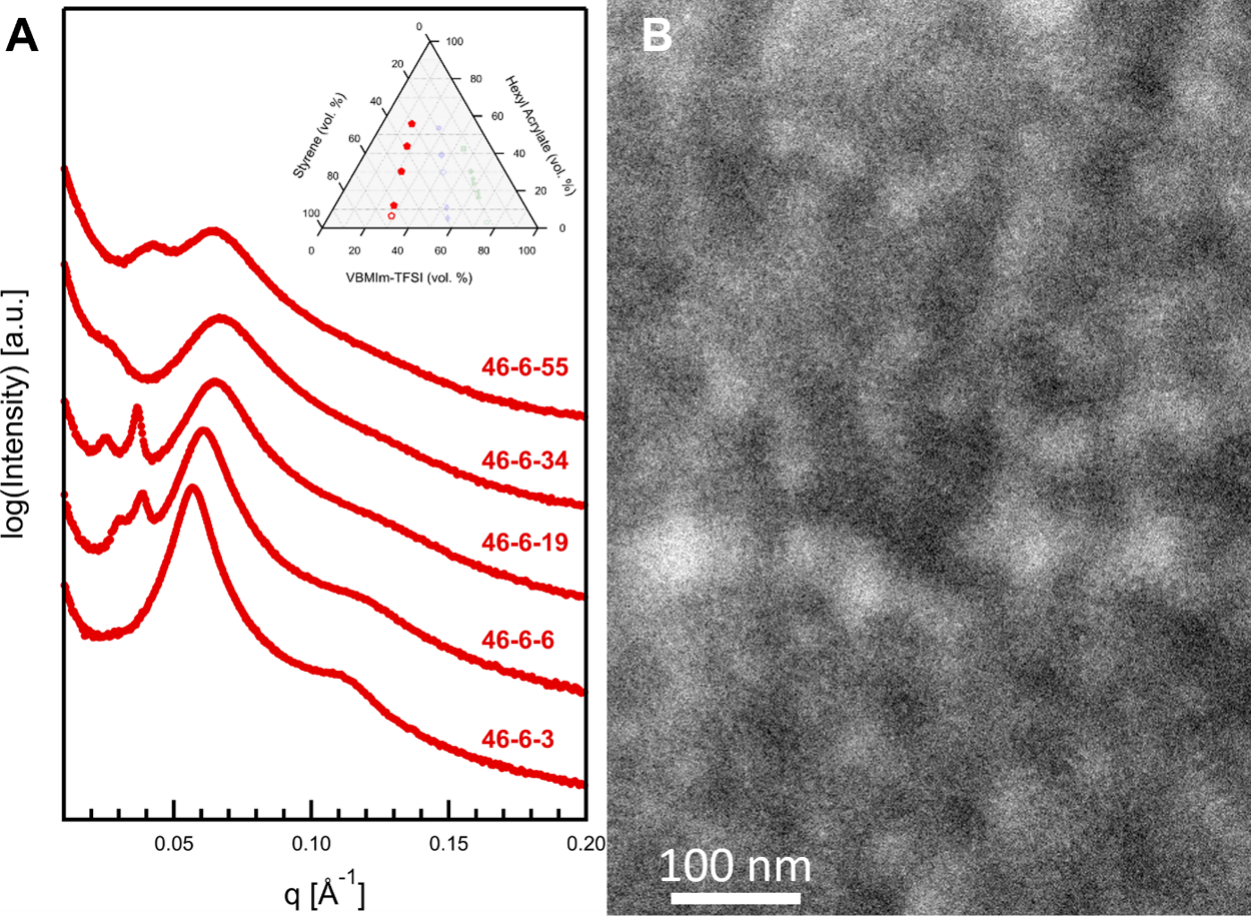
(A) Vertically scaled SAXS of 46–6–3 through 46–6–55, and (B) a HAADF-STEM micrograph of 46–6–19, showing a microphase separated morphology. Inset phase diagram indicates samples described in this figure.

SAXS and HAADF-STEM data for poly(S-*b*-VBMIm-TFSI-*b*-HA) polymers containing six repeat units of VBMIm-TFSI are shown in Figure 5. Sample 46–6–3 with the lowest HA content shows two Bragg diffraction peaks including a substantial scattering maximum centered at 0.0569 Å^–1^ and a shoulder at 0.108 Å^–1^. An increase in HA content from 6 vol % (46–6–3) to 12 vol % (46–6–6) results in a slight shift to higher *q* of the primary scattering maximum and shoulder, and the emergence of two scattering peaks at lower *q*. Increasing HA content from 12 vol % (46–6–6) to 30 vol % (46–6–19) results in another slight shift of the primary maximum and shoulder to higher *q*, while the two lower-*q* peaks separate and shift to slightly lower angles. Increasing HA again to 44 vol % (46–6–34) further shifts the primary maximum and shoulder to higher *q*, 0.0672 Å^–1^, with the two low-*q* features replaced by a single, broad feature at 0.0280 Å^–1^. The highest HA content polymer in this series, 46–6–55, continues to show a strong maximum and shoulder, now shifted back to slightly lower *q* (0.0647 Å^–1^).

TEM data for 46–6–19 is shown in Figure 5B. A small feature that is light in color relative to the surrounding material can be observed throughout the micrograph, with a typical distance between features of roughly 10 nm. This suggests microphase separated domains comprising the short VBMIm-TFSI blocks. No overall organization of the domains is observed, consistent with the lack of higher order Bragg diffraction peaks in the SAXS data. A large-scale fluctuation in color on a large length scale, roughly 70–100 nm, is visible but was also observed in other samples having different VBMIm-TFSI contents, suggesting that this may be an artifact of the microtomy process. To improve contrast for TEM, negative staining with OsO_4_ was performed on select samples, but was found to reduce contrast by preferentially staining the S block.

The morphological behavior of samples 46–6–3, 46–6–6, 46–6–19, 46–6–34, and 46–6–55 is not definitively discernible from the SAXS or available TEM data. The TEM data suggest a microphase separated morphology of PIL domains in a matrix of S and/or HA, such as is commonly observed in ion-containing polymers. The primary scattering maxima and shoulder for 46–6–3 and 46–6–6 suggest the possibility of a lamellar morphology, but a detailed fit of the data do not support that interpretation (see Supporting Information Figure S5). However, the corresponding *d*-spacing for the highest intensity peak for those samples (e.g., 11.0 nm for 46–6–3, 9.5 nm for 46–6–19) approximately agrees with the observed separation between microphase-separated domains in TEM.

The origin of the lower angle peaks observed in all but 46–6–3 is also unclear. In 46–6–19, for example, the peak positions correspond to correlation lengths of 21.0 and 17.3 nm, distances which are not related to discernible features in the TEM micrograph. It is notable that the scattering features of the possible microphase-separated morphology do not change as the volume fraction of HA increases, but simply shift to higher angles. This suggests that the HA is not incorporating into the S/VBMIm-TFSI structure, but rather being excluded. In that case, the lower-*q* features in the SAXS data may be domains of HA in a matrix of S/VBMIm-TFSI.

**Figure 6:**
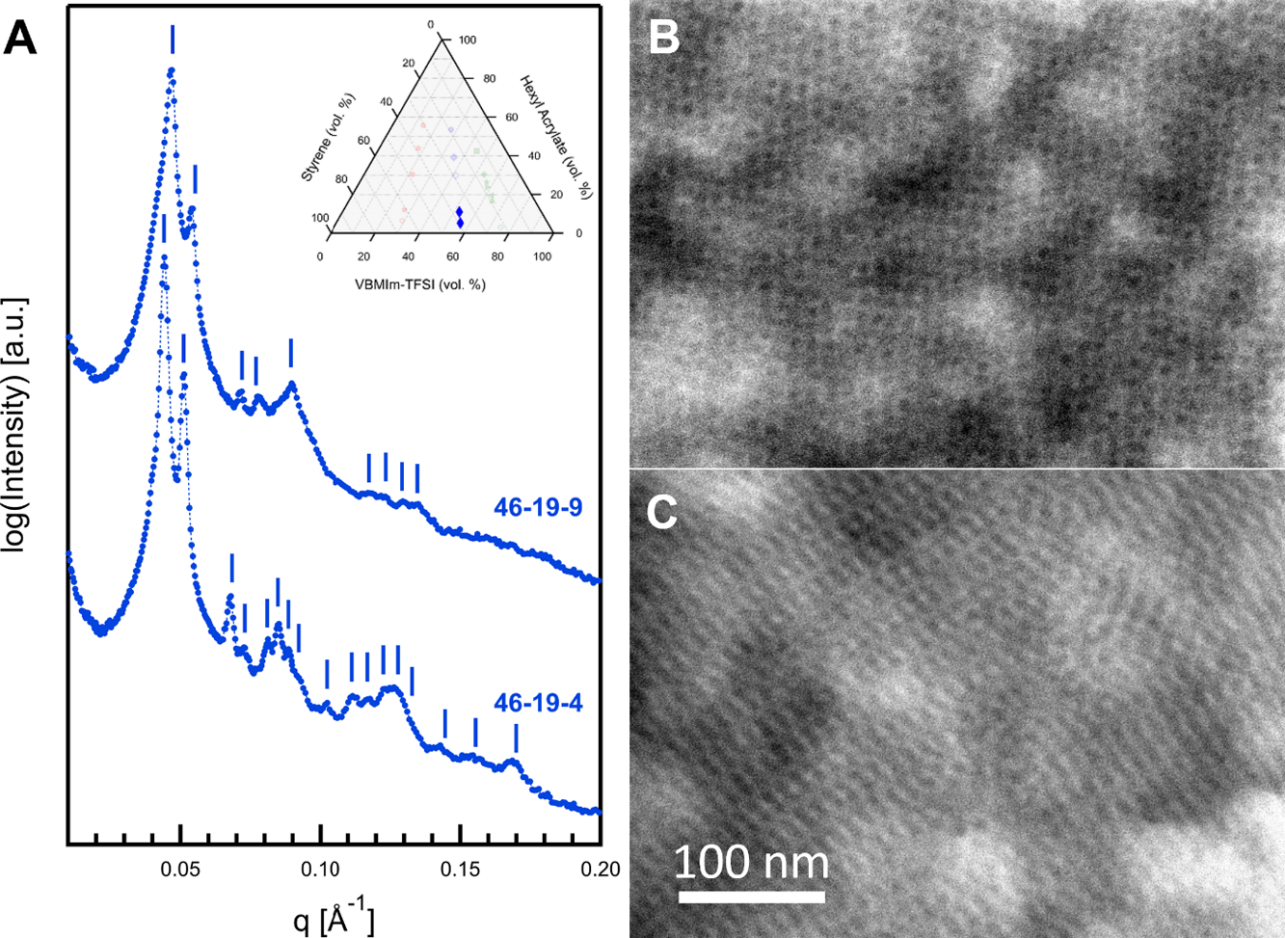
(A) Vertically scaled SAXS data for 46–19–4 and 46–19–9, and (B, C) HAADF-STEM data for 46–19–4. Both samples appear to form the Q^230^ gyroid morphology.

In the next group of polymers, the length of the VBMIm-TFSI midblock was increased from 6 repeat units to 19 repeat units. SAXS and TEM data for the first two samples in this set, 46–19–4 and 46–19–9, are shown in Figure 6. Both samples produced near single-crystal scattering with individual diffraction peaks rather than rings, indicating the presence of exceptional long-range order. After azimuthal averaging, the 1D SAXS data for 46–19–4 were found to have at least 15 discernible diffraction peaks. The peaks were observed at *q*:q* ratios of , , , , , , , , , , , , , , , , , and . These correspond to the Q^230^ gyroid, as previously reported in several other triblock terpolymer systems.^41,71,72^ Some predicted Q^230^ reflections were not observed, which may be due to disorder or SAXS resolution limitations due to the pixel size of the detector. The observed peak ratios and absence of specific reflections eliminate other similar morphologies, including the O^70^ network (for which the *q*:q* begins with 1, 3.57, and 4) and the Q^214^ alternating gyroid (for which the first three reflections are expected at *q*:q* ratios of , , and ). The SAXS data for 46–19–9 were also found to be a good match for the Q^230^ gyroid, with Bragg diffraction maxima identified at *q*:q* ratios of , , , , , , , , , , and . Figure 6B shows a representative HAADF-STEM micrograph from 46–19–4. Although faint, the characteristic “wagon wheel” structure of the Q^230^ gyroid morphology is visible and was found to have long-range order consistent with the numerous reflections observed in SAXS. Figure 6C is reminiscent of micrographs reported by Hückstädt and co-workers^40^ to which they attributed the (112) projection of the Q^230^ gyroid. As noted before, efforts to enhance the contrast in these materials by negative staining were unsuccessful due to the preference of OsO_4_ stain for the double bonds of the polystyrene phenyl ring.

**Figure 7:**
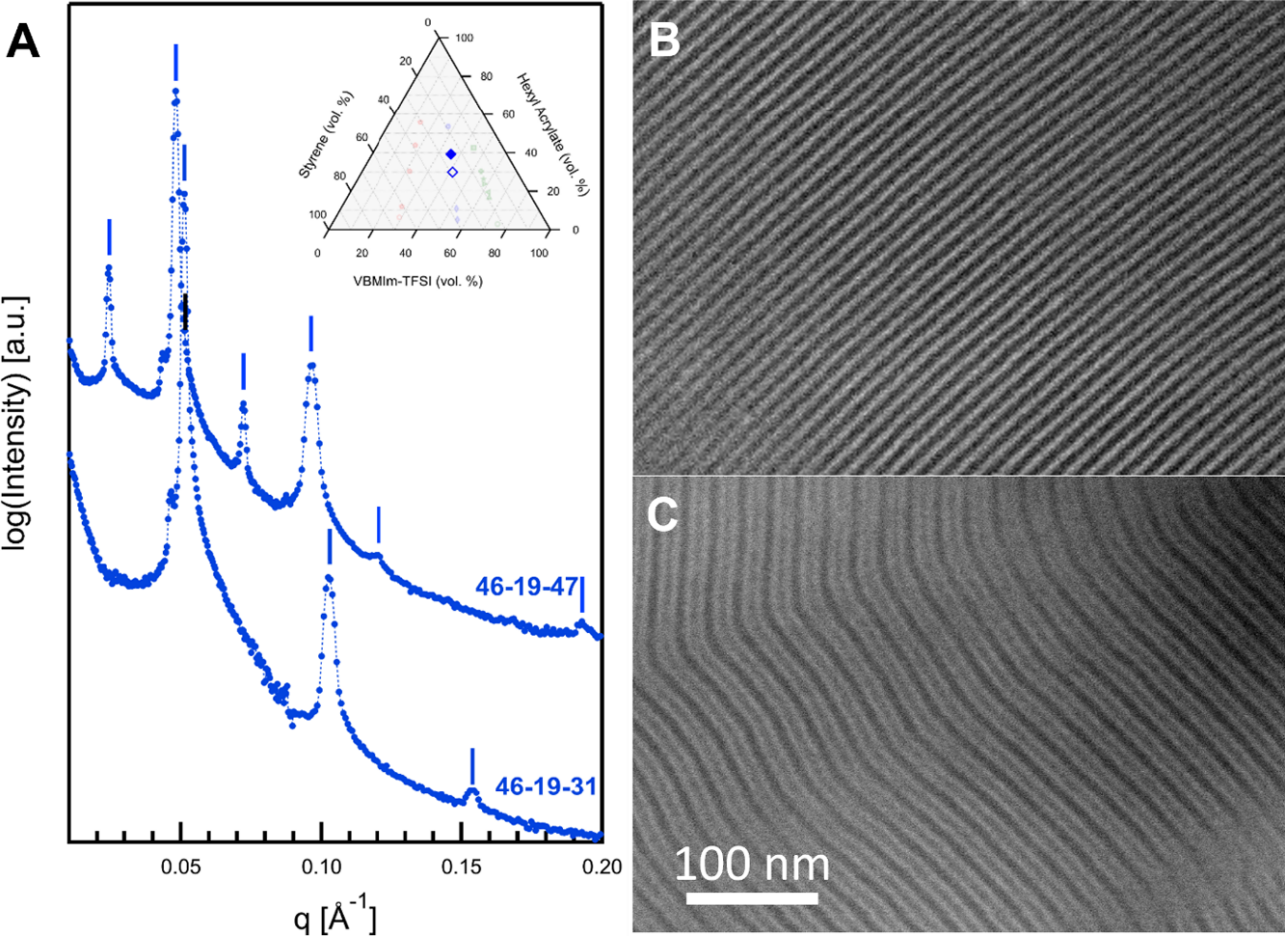
(A) Vertically scaled SAXS data for 46–19–31 and 46–19–47, (B) HAADF-STEM micrograph for 46–19–31, and (C) HAADF-STEM micrograph collected from 46–19–47. Sample 46–19–31 shows a two-phase lamellar morphology (L_2_), while 46–19–47 shows a three-phase lamellar morphology of the ABCB type (L_3_).

Figure 7 shows the SAXS and HAADF-STEM data collected for 46–19–31 and 46–19–47. 46–19–31 forms a two-phase lamellar morphology (L_2_), with alternating layers of VBMIm-TFSI (bright in HAADF-STEM, Figure 7B) and what appears to be a single-phase mixture of S and HA. Bragg diffraction peaks are observed at *q*:q* ratios of 1, 2, 3, and 4 (Figure 7A), where q* is assigned to the first observed peak. The diffraction peaks correspond to a lamellar morphology with *d*-spacing of 12.6 nm. 46–19–47 shows a three-phase lamellar structure (L_3_) of the kind previously observed in certain highly frustrated triblock terpolymer systems.^45,46,73−75^ Bragg diffraction peaks were observed at *q*:q* ratios of 1, 2, 3, 4, 5, and 8, where q* is assigned to the first observed peak. Here, each block of the ABC terpolymer has microphase separated into separate domains, and the domains repeat in the order ABCB. This results in alternating domains of S and HA separated by VBMIm-TFSI domains. In the HAADF-STEM micrograph shown in Figure 7C, due to differences in mean free path of each block, the midblock VBMIm-TFSI appears bright, S appears darkest, and HA is a shade of gray between VBMIm-TFSI and S. The SAXS data in Figure 7A support this analysis: the high X-ray scattering contrast between VBMIm-TFSI and both S and HA immediately suggests that the highest intensity peak, observed at 0.048 Å^–1^ and corresponding to a lamellar period of 13.0 nm, can be attributed to the period of the VBMIm-TFSI domains. The primary diffraction maximum, observed at 0.0241 Å^–1^ and corresponding to a lamellar period of 26.1 nm, is therefore attributed to both the S domains (occurring every 26.1 nm after a sequence of VBMIm-TFSI, HA, and VBMIm-TFSI layers) and the HA domains (occurring every 26.1 nm after a sequence of VBMIm-TFSI, S, and VBMIm-TFSI layers).

**Figure 8:**
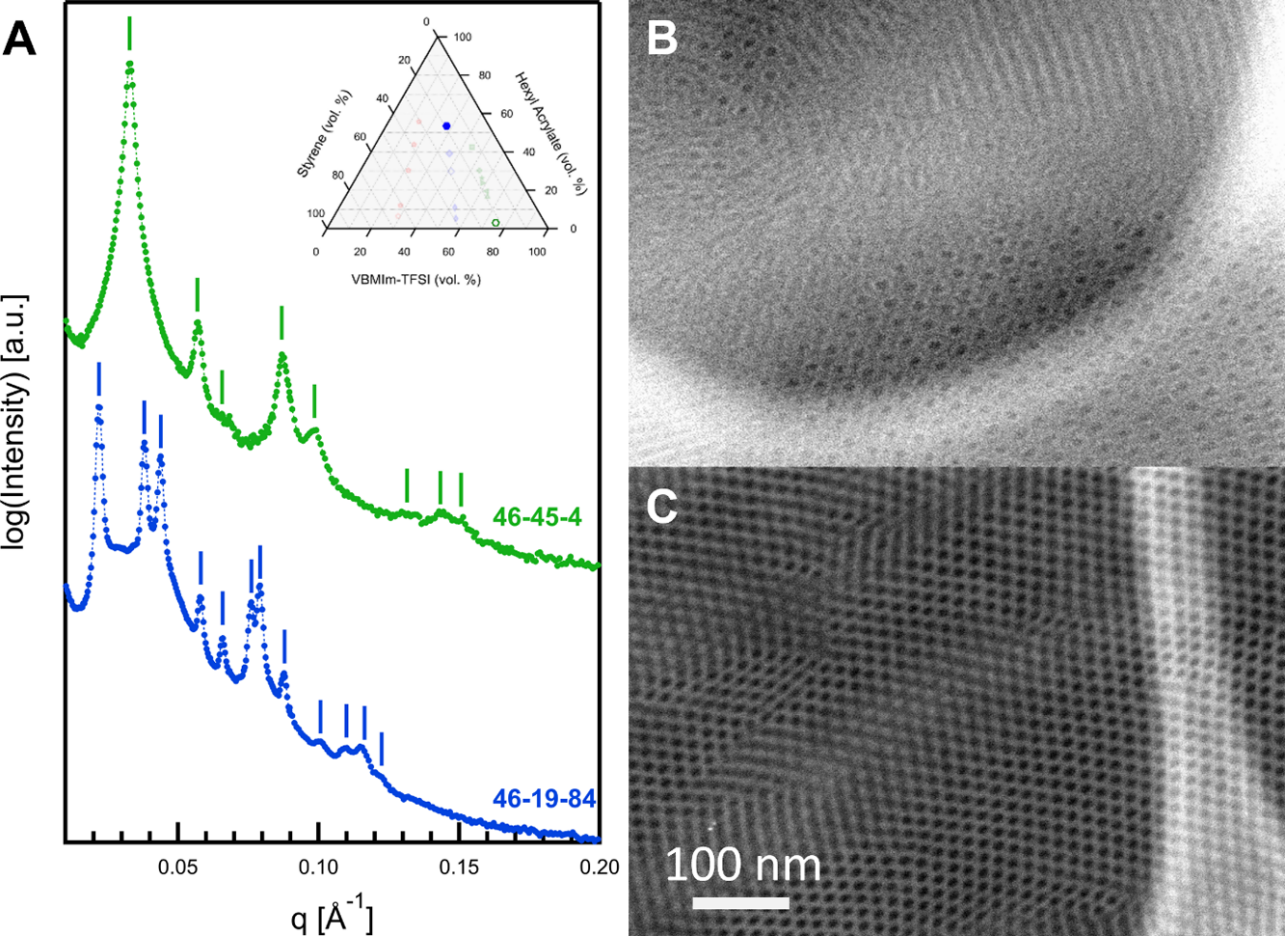
(A) Vertically scaled SAXS data for 46–19–84 (blue) and 46–45–4 (green), and HAADF-STEM micrographs for (B) 46–19–84 and (C) 46–45–4. Sample 46–19–84 has a three-phase morphology of hexagonally packed core–shell cylinders (C_CS_), while sample 46–45–4 shows a two-phase morphology of hexagonally packed cylinders (C_2_).

Figure 8 shows the SAXS and HAADF-STEM data collected for 46–19–84 and 46–45–4. These two polymers fall into very different regions of the ternary phase diagram (Figure 3) due to the very different VBMIm-TFSI (ϕ_PIL_ of 27% for 46–19–84, and ϕ_PIL_ of 75% for 46–45–4) and HA (ϕ_HA_ of 54% for 46–19–84, and ϕ_HA_ of 3% for 46–45–4) contents. The SAXS data for 46–45–4 shows multiple Bragg diffraction peaks and matches by predicted scattering from a morphology of cylinders on a hexagonal lattice. Given the very low HA content, this is most likely a two-phase morphology of S cylinders in VBMIm-TFSI (C_2_). Assigning the primary diffraction peak to the (10) reflection, one finds peaks at predicted locations for *q*:q* ratios of , , , , , , and . This fit corresponds to a *d*-spacing for the (10) plane of 19.1 nm and a hexagonal lattice parameter, *a*, of 22.1 nm.^76^ Reflections for *q*:q* ratios of  and  are absent, likely extinguished by a minimum in the cylinder form factor. The HAADF-STEM data for 46–45–4 (Figure 8C) supports the assignment of C_2_, showing dark circular regions on a 2D hexagonal lattice with a lighter material as matrix, corresponding well to the predicted intensities for HAADF-STEM using mean free path calculations. The lacey carbon support film on the TEM grid is also visible in the right portion of Figure 8C.

The SAXS data for 46–19–84 is more complex. In this case, 12 Bragg diffraction peaks can be discerned, indicating very good long-range order. For a 2D hexagonal lattice, after assigning the primary diffraction peak at 0.0220 Å^–1^ to the (10) reflection, observed peaks agree with predicted spacings for *q*:q* ratios of , , , , , , , , , , and , confirming a morphology of hexagonally packed cylinders. The TEM data in Figure 8B reveal that the morphology is one of core–shell cylinders on a 2D hexagonal lattice. A core of S is still observed (dark), but now the core is surrounded by a shell of VBMIm-TFSI (light), and the matrix is HA (intermediate).

**Figure 9:**
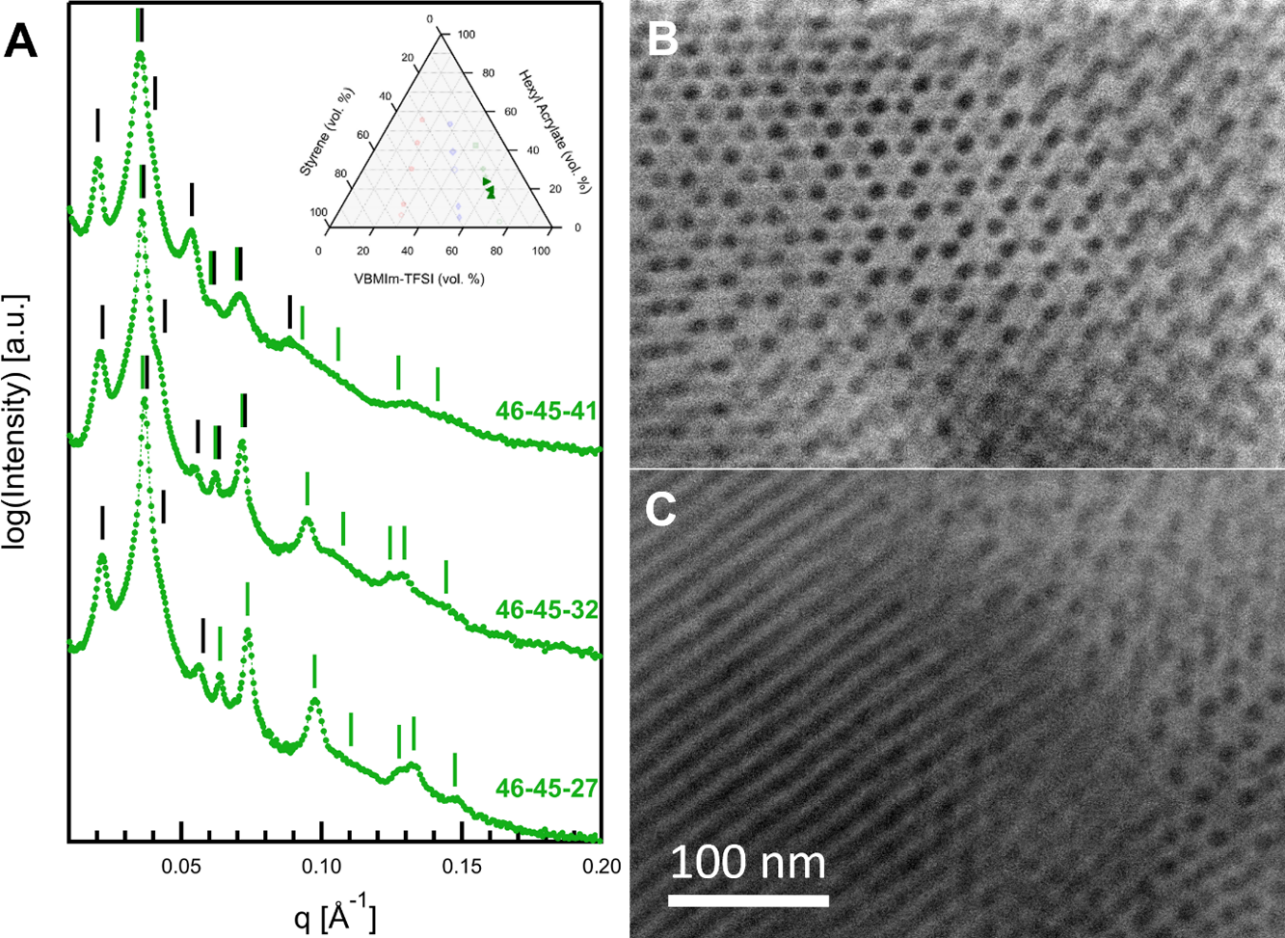
(A) Vertically scaled SAXS data for samples 46–45–27, 46–45–32, and 46–45–41. (B, C) HAADF-STEM data for 46–45–41. These three samples all showed a superlattice morphology of hexagonally packed cylinders (C_SL_). In the SAXS data, the black lines correspond to diffraction peaks from the larger lattice, while the green lines mark those from the smaller lattice.

The morphological behavior of 46–45–27, 46–45–32, and 46–45–41 is quite complex and difficult to describe based on the SAXS data alone. As seen in Figure 9A, the data for these three samples are all similar, and the same morphology was expected for each sample given the small differences in composition. The SAXS data again show a low-*q* peak with lower intensity than the second observed diffraction peak, suggesting a three-phase morphology. In this case, it was found that the data indicate the formation of a superlattice of cylinders on two superimposed 2D hexagonal lattices, such as that observed by Brinkman and co-workers.^77^ The primary scattering maximum for 46–45–27, located at 0.0218 Å^–1^, corresponds to a 2D hexagonal lattice with *d*-spacing for the (10) reflection of 28.7 nm, and a 2D hexagonal lattice parameter, *a*, of 33.1 nm. Bragg diffraction peaks for this lattice are observed at *q*:q* ratios of approximately , , and , indicated in Figure 9A by black lines over the peak positions. Note that the  reflection is only a shoulder on the strong peak at 0.037 Å^–1^. For the smaller lattice, the primary diffraction peak is observed at 0.037 Å^–1^. The *d*-spacing of this refection is 17.0 nm, corresponding to a lattice parameter, *a*, of 19.7 nm. Higher order diffraction peaks are observed at *q*:q* ratios of , , , , and , indicated by green lines over the observed and predicted peak positions. The highest intensity peak is formed by the superposition of the second-order peak of the larger morphology and the primary diffraction peak from the smaller morphology. HAADF-STEM micrographs in Figure 9B,C confirm the formation of this superlattice morphology. In Figure 9B, the origin of the superlattice structure can be seen as the formation of either S (dark) or HA (gray) cylinders in a VBMIm-TFSI (light) matrix. Figure 9C confirms the cylindrical nature of the morphology when viewed normal to the cylinder long axis.

**Figure 10:**
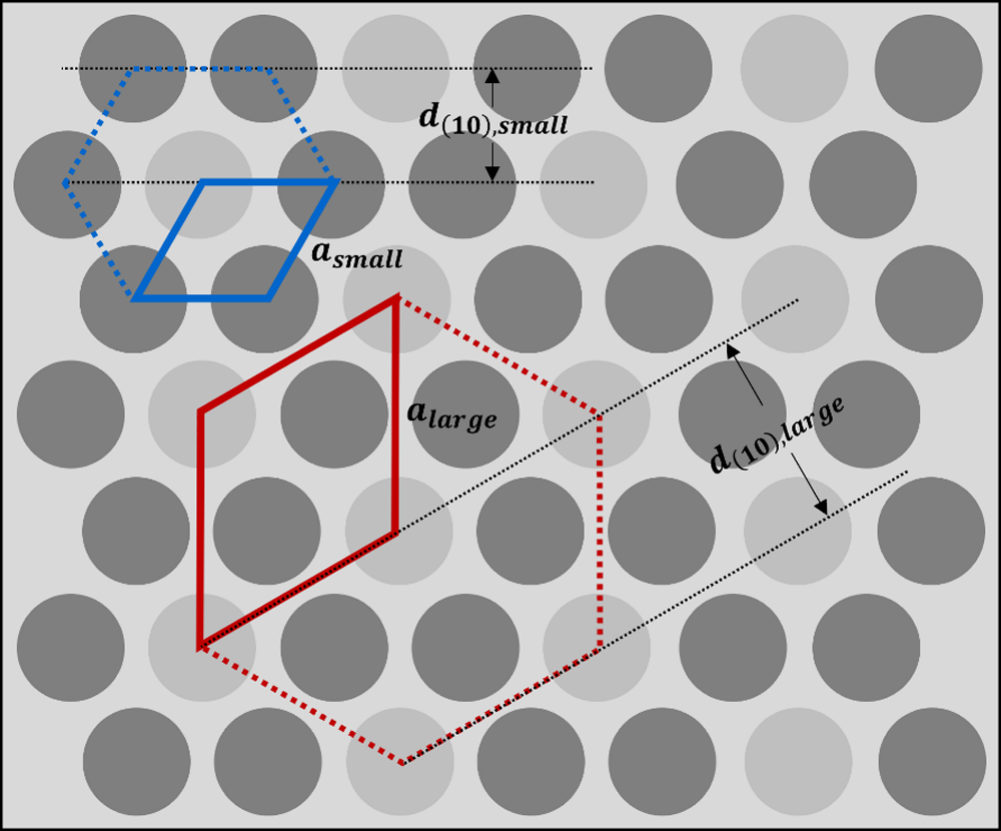
Schematic of a superlattice of hexagonally packed cylinders, viewed along the cylinder axis. Domain shadings correspond to mean free path calculations (S is dark gray, HA is intermediate gray, and VBMIm-TFSI is light gray). The blue trapezoid marks the primitive unit cell for the smaller hexagonal structure, while the red trapezoid shows the primitive unit cell for the larger hexagonal structure. The (10) plane spacings for the large and small lattices, *d*_(10),large_ and *d*_(10),small_, are indicated, as are the lattice parameters, *a*_small_ and *a*_large_.

This superlattice morphology is illustrated in Figure 10. The smaller morphology gives rise to lattice positions that form a 2D hexagonal structure. However, instead of all cylinders comprising a single component (i.e., S), here some of the cylinders are replaced with another component (HA). The remaining block of the ABC triblock (i.e., VBMIm-TFSI) forms the matrix. This also illustrates the relationship between the lattice parameters of the two matrices, where the lattice parameter of the larger lattice (*a*_large_) is twice the *d*-spacing of the (10) plane in the smaller lattice (*d*_(10),small_).

The SAXS data for 46–45–32 and 46–45–41 are also explained by this morphology. For 46–45–32, the primary diffraction peak is observed at 0.0213 Å^–1^, with higher order reflections at *q*:q* ratios of approximately , , ,  and . This scattering corresponds to a *d*_(10)_ of 29.6 nm and a lattice parameter of 34.1 nm. The primary diffraction peak for the smaller lattice, of S domains in a VBMIm-TFSI matrix, is observed at 0.0359 Å^–1^. This corresponds to *d*_(10)_ of 17.5 nm and a lattice parameter of 20.2 nm. Higher order reflections are observed at *q*:q* ratios of , , , , , , and . Overlap of the Bragg diffraction peaks is observed for the first three diffraction maxima of the smaller lattice, as noted in Figure 9A. For 46–45–41, the primary diffraction peak is observed at 0.0204 Å^–1^, with higher order diffraction peaks observed at *q*:q* ratios of , , , , , and . This diffraction pattern indicates that the *d*-spacing for the (10) plane of the large lattice of HA cylinders in a matrix of VBMIm-TFSI and S cylinders is 30.9 nm and that the lattice parameter is 35.6 nm. The primary diffraction peak for the smaller lattice of S cylinders in a VBMIm-TFSI matrix is observed at 0.0354 Å^–1^, corresponding to *d*_(10)_ of 17.8 nm and a lattice parameter of 20.5 nm. Higher order reflections are visible at *q*:q* ratios of , , , , and . The higher order peak assignments in this case are tenuous. The weak feature observed at 0.13 Å^–1^ could be attributed to a form factor fringe given that the intermediate reflections are absent.

**Figure 11:**
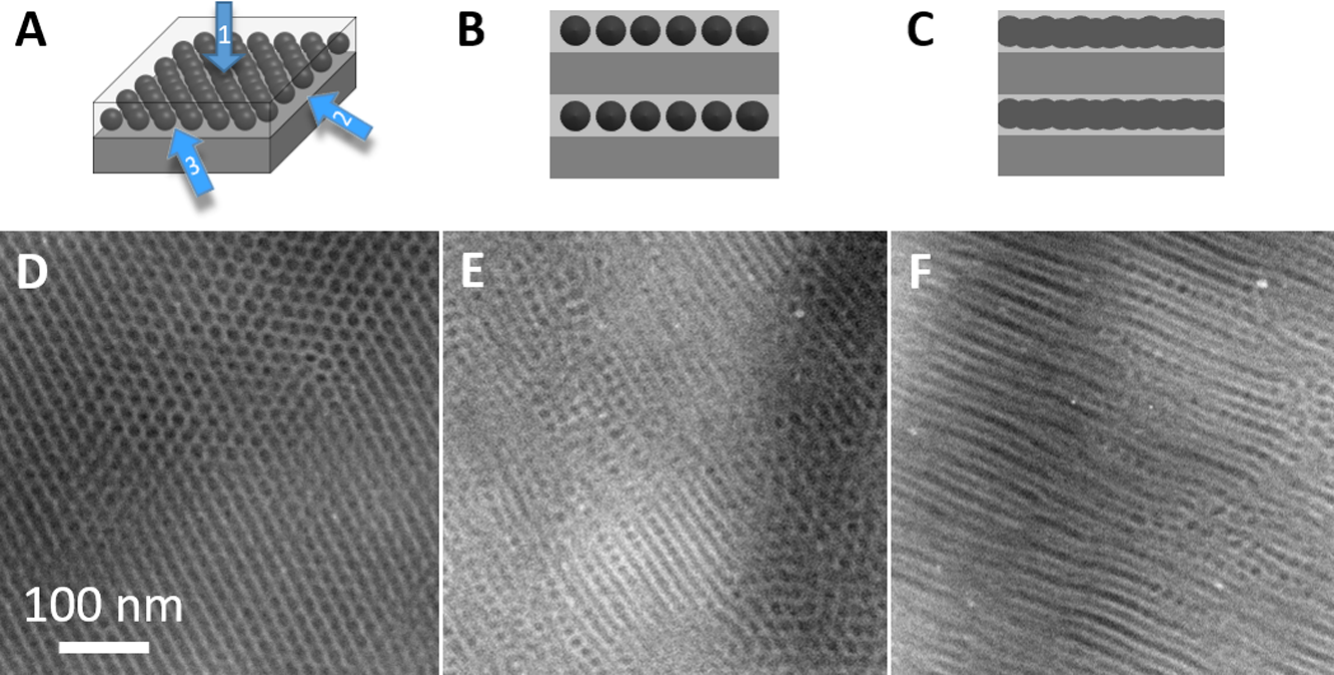
Schematics showing views of the L_SI_ morphology in different projections (A: overall scheme; B: scheme from arrow 2; C: scheme from arrow 3), and corresponding HAADF-STEM micrographs from 46–45–57 showing the observed morphologies (D: micrograph viewed from arrow 1; E: micrograph viewed from arrow 2; F: micrograph viewed from both arrow 2 and arrow 3).

Increasing the HA content further, from 24 vol % (46–45–41) to 26 vol % (46–45–47) and 30 vol % (46–45–57), nudges the morphological behavior into a related but substantially different structure. As illustrated in Figure 11, this morphology appears to be a type of spheres-in-lamellae (L_SI_). This morphology is predicted to form in highly frustrated ABC triblock terpolymer systems, where the B block is much less miscible with the A and C blocks than A and C are from each other.^38^

Figure 11A shows a schematic of the L_SI_ morphology. In this morphology, one end block (HA) and the midblock (VBMIm-TFSI) form a lamellar morphology, while the S block microphase separates into spheres within the VBMIm-TFSI lamellae. In the schematic, the spheres are shown forming a 2D hexagonal lattice (Figure 11A–C). This morphology, when viewed normal to the plane of the lamellae (arrow 1), would show the hexagonal packing of dark domains in a lighter matrix. When viewed in the plane of the lamellae and along one of the hexagonal lattice axes (arrow 2), as illustrated in Figure 11B, the lamellar structure is clearly visible and the alignment of the view with the hexagonal lattice allows the separation between spheres to be observed. When viewed in the plane of the lamellae, but in a direction substantially different from a hexagonal lattice axis (arrow 3), the projected image in a TEM would be expected to resemble that illustrated in Figure 11C. Now the lamellae comprising block B and spheres of A appear as a layer of A sandwiched between layers of B, very similar to the ABCB lamellar structure observed for 46–19–47 (Figure 7C). Figure 11D–F shows the TEM images of 46–45–57, which is a representative of the morphologies illustrated above. In Figure 11D, the hexagonal structure predicted for viewing along arrow 1 is observed, with dark domains (likely S) forming a 2D hexagonal lattice in a light matrix (a stack of VBMIm-TFSI and HA). In Figure 11E, regions where the view aligns with arrow 2 in the schematic, S domains (darkest) are visible in layers of VBMIm-TFSI (lightest), alternating with HA (intermediate gray) layers. Figure 11F shows additional regions where the view is aligned with the hexagonal lattice (arrow 2), but it also clearly shows the view predicted to be observed along arrow 3 and illustrated in Figure 11C.

**Figure 12:**
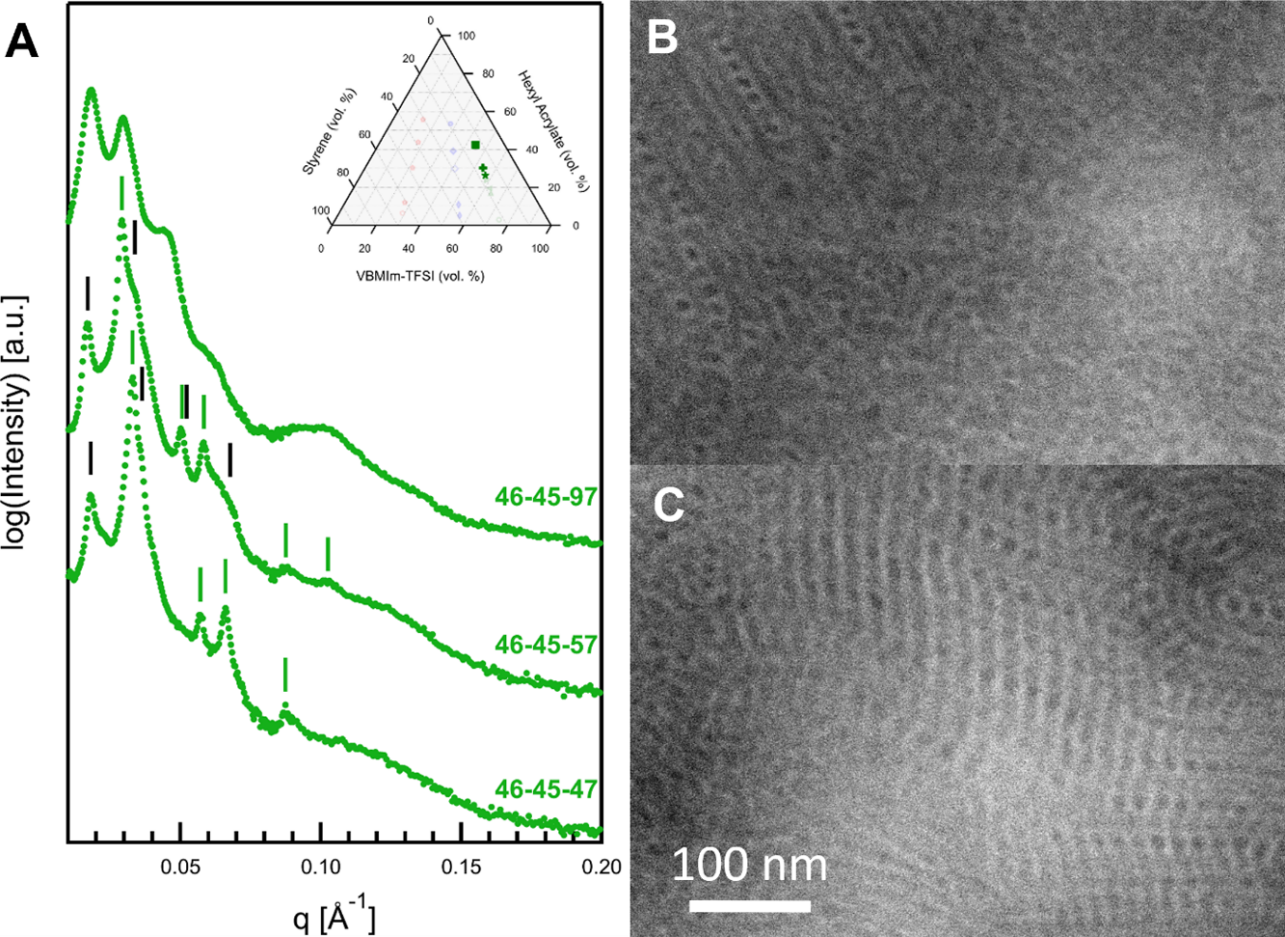
(A) Vertically scaled SAXS data for 46–45–47, 46–45–57, and 46–45–97. (B, C) HAADF-STEM micrographs for 46–45–97, showing the spheres-in-lamellae (L_SI_) morphology. In (A), the black lines represent lamellar diffraction peak positions, and the green lines indicate diffraction peaks from the spherical morphology.

The SAXS data for 46–45–57, shown in Figure 12A, support this analysis. Although visually similar to the SAXS data in Figure 9, where a morphology of cylinders on a superlattice was assigned, the primary diffraction peak observed here generally fits a lamellar structure, while the highest intensity peak and higher order reflections confirm the hexagonal packing (of S spheres). With this assignment for the primary diffraction peak (0.0170 Å^–1^), a diffraction peak at 2q* (0.0364 Å^–1^) accounts for the shoulder observed on the strong diffraction peak centered at 0.0292 Å^–1^. Diffraction peaks from the lamellar structure predicted at *q*:q* ratios of 3, 4, and 6 are also observed. The assignment of the strong peak at 0.0292 Å^–1^ to the hexagonal lattice of S spheres results in good agreement between the data and predicted diffraction maxima at , , , and , with the absence of a reflection of . The SAXS data for 46–45–47, also shown in Figure 12A, can be fit in the same way. The lowest angle peak, at 0.0182 Å^–1^, is assigned to a lamellar structure. The 2q* peak from the lamellar structure is predicted to occur at 0.0364 Å^–1^ and matches well with the shoulder observed on the most intense peak in the pattern. The most intense peak, centered at 0.0330 Å^–1^, is assigned to a 2D hexagonal array of S spheres occurring within the VBMIm-TFSI matrix. Predicted diffraction peaks are observed at *q*:q* ratios of , , and , fitting the experimental data very well.

Finally, Figure 12 also shows the SAXS and HAADF-STEM data collected for 46–45–97. Although the SAXS data contain several clear maxima, no single morphology or combination of morphologies successfully predicts the scattering data. The TEM data illustrate the origin of this confusing information. Most of the sample appears to have a microphase-separated, but disordered structure, such as that visible in the lower right portion of Figure 12B. Other regions appear to show an L_SI_ morphology (Figure 12B top left). Still other regions show a morphology that appears to be a combination of a lamellae of VBMIm-TFSI and lamellae which comprise domains of S and HA (Figure 12C). The most likely explanation for these data is that the sample is far from equilibrium, with local variations in morphology reflecting local variations in composition and kinetically trapped morphologies. It is worth noting that this sample has the highest molecular weight and dispersity of the series of 17 polymers. The morphology, domain spacing, and peak locations of SAXS are summarized in Table 2.

**Table 2:** Morphology, Domain Spacing (d*), and SAXS Peak Locations of Poly(S-*b*-VBMIm-TFSI-*b*-HA)

polymer	morphology^a^	d* (nm)	lattice parameter (nm)	Bragg diffraction peak locations^b^
46–6–3	D_2_	11.0	N/A	1, 1.89
46–6–6	D_3_	10.3	N/A	1, 1.27, 2, 3.20
46–6–19	D_3_	9.5	N/A	1, 1.40, 2.51, 3.85
46–6–34	D_3_	9.3	N/A	1, 1.39
46–6–55	D_3_	9.6	N/A	1, 1.60
46–19–4	Q^230^	14.2	34.7	√6, √8, √14, √16, √20, √22, √24, √26, √32, √38, √42, √46, √50, √54, √64, √74, √80, √88
46–19–9	Q^230^	13.5	32.9	√6, √8, √14, √16, √22, √24, √26, √38, √42, √46, √50
46–19–31	L_2_	12.6	12.3	1, 2, 3, 4
46–19–47	L_3_	13.0	13.0	1, 2, 4
		26.1	26.1	1, 2, 3, 4, 5, 8
46–19–84	C_CS_	28.5	33.0	1, √3, √4, √7, √9, √12, √13, √16, √21, √25, √28, √31
46–45–4	C_2_	19.1	22.1	1, √3, √4, √7, √9, √16, √19, √21
46–45–27	C_SL_	28.7	33.1	1, √3, √4, √7,
		17.0	19.7	1, √3, √4, √7, √9, √12, √13, √16
46–45–32	C_SL_	29.6	34.1	1, √3, √4, √7, √9, √12
		17.5	20.2	1, √3, √4, √7, √9, √12, √13, √16
46–45–41	C_SL_	30.9	35.6	1, √3, √4, √7, √9, √12, √19
		17.8	20.5	1, √3, √4, √7, √9, √13, √16
46–45–47	L_SI_	34.5	34.5	1, 2
		19.0	22.0	1, √3, √4, √7
46–45–57	L_SI_	36.8	36.8	1, 2, 3, 4, 6
		21.5	24.8	1, √3, √4, √9, √12
46–45–97	L_SI_*	34.5	N/A	1, 1.63, 2.41, 3.40, 5.47

a Acronyms: D_2_, two-phase disordered microphase separation; D_3_, three-phase disordered microphase separation; C_2_, two-phase hexagonally packed cylinders; C_CS_, core–shell hexagonally packed cylinders; C_SL_, hexagonal superlattice; Q^230^, core–shell double gyroid; L_2_, two-domain AB lamellae; L_3_, three-domain ABCB lamellae; and L_SI_, spheres in lamellae.

b Observed Bragg peak locations are represented as the multiple of q* (the primary peak position in the SAXS profile). Note that the identified reflections and their corresponding domain spacing were listed into two rows where there are 2 morphologies superimposed.

### Morphology Discussion

As discussed in the Introduction, one factor that significantly influences the phase behavior of ABC triblock terpolymers is the Flory–Huggins segmental interaction parameter χ. For the materials studied here, explicit values of χ are not available. We are not aware of a study on the miscibility of PS and PHA, but PS and poly(methyl methacrylate) are well-known to be mildly immiscible (χ = 0.0194 at 25 °C), and poly(n-pentyl methacrylate) is also mildly immiscible with deuterated PS (χ = 0.0180 at 25 °C).^78^ Using group contribution methods, the solubility parameters for PS, PHA, and poly(methyl methacrylate) (PMMA) were calculated to be 17.0, 18.5, and 20.0 MPa^1/2^, respectively, further suggesting that PHA and PS are weakly immiscible.^79^ However, it is known that the Coulombic interactions in ion-containing polymers can dominate their phase behavior by creating a strong driving force for association.^80,81^ Combined with the observed phase behavior described above, we conclude that the ABC PIL triblock terpolymers in this work are frustrated. The observation of spheres-in-lamellae suggests that χ_BC_ ≥ χ_AB_ ≥ χ_AC_, placing these polymers in the third category of “highly frustrated”. Furthermore, the observation of PS spheres in the PVBMIm-TFSI layers of the PVBMIm-TFSI/PHA lamellar morphology suggests that VBMIm-TFSI/HA interaction is very strongly unfavorable, such that χ_VBMIm-HA_ is larger than χ_VBMIm-S_. The connectivity of the PS block to the PVBMIm-TFSI block forces the system to form PVBMIm-TFSI/PS interfaces. Therefore, we surmise that the order of χ parameters is χ_VBMIm-HA_ ≥ χ_VBMIm-S_ ≥ χ_S-HA_. This order of miscibility parameters is supported by other data, including the morphological behavior of samples 46–6–3 through 46–6–55 in which a small increase in the HA content, from 6 to 12 vol % is enough to drive the system to form longer-range microphase separated domains in addition to the S-VBMIm morphology of 46–6–3.

A second factor that must be considered for the materials in this study is the effect of the synthesis strategy on the relevant molecular characteristics of the PIL triblock terpolymers. It is well established that the driving force for microphase separation can be divided into different regimes based on the segmental interaction parameter, χ, and the degree of polymerization, N.^82,83^ When χ*N* is near but greater than the order–disorder transition, the polymer is said to be in the weak segregation regime. In this regime, the enthalpic benefit for microphase separation is only slightly greater than the loss of entropy incurred. As χ*N* increases, the driving force for microphase separation increases and the polymer is said to be in the intermediate segregation regime. For sufficiently large *N*, the materials are said to be in the strong segregation limit, at which point morphology becomes stable, no longer affected by small changes in *N* or in temperature. The triblock terpolymers in this study range in molecular weight from approximately *M*_n_ of 8.4 kDa (46–6–3) to *M*_n_ of 42 kDa (46–45–97). The values of *N*, therefore, are very low. The observation of microphase separation in the lowest *M*_w_ materials indicates that the segmental interaction parameters must be substantial. It is also noteworthy that the lowest molecular weight samples are likely not entangled. The entanglement molecular weight of neat PS, for example, is 19 kDa,^84−86^ roughly four times greater than the PS block *M*_w_ in 46–6–3.

A final molecular factor affecting morphological behavior is the dispersity, *Đ* = *M*_w_/*M*_n_, as listed in Table 1. Where most block copolymers studied for the purpose of understanding phase behavior have very low *Đ*, typically 1.05 or lower, the materials studied here have *Đ* ranging from 1.19 to 1.81. Although there is some evidence that dispersity can aid self-assembly behavior in polymers,^87^ in general it is understood that complex structures are best formed when comprised of narrow size-disperse components.^88^

**Figure 13:**
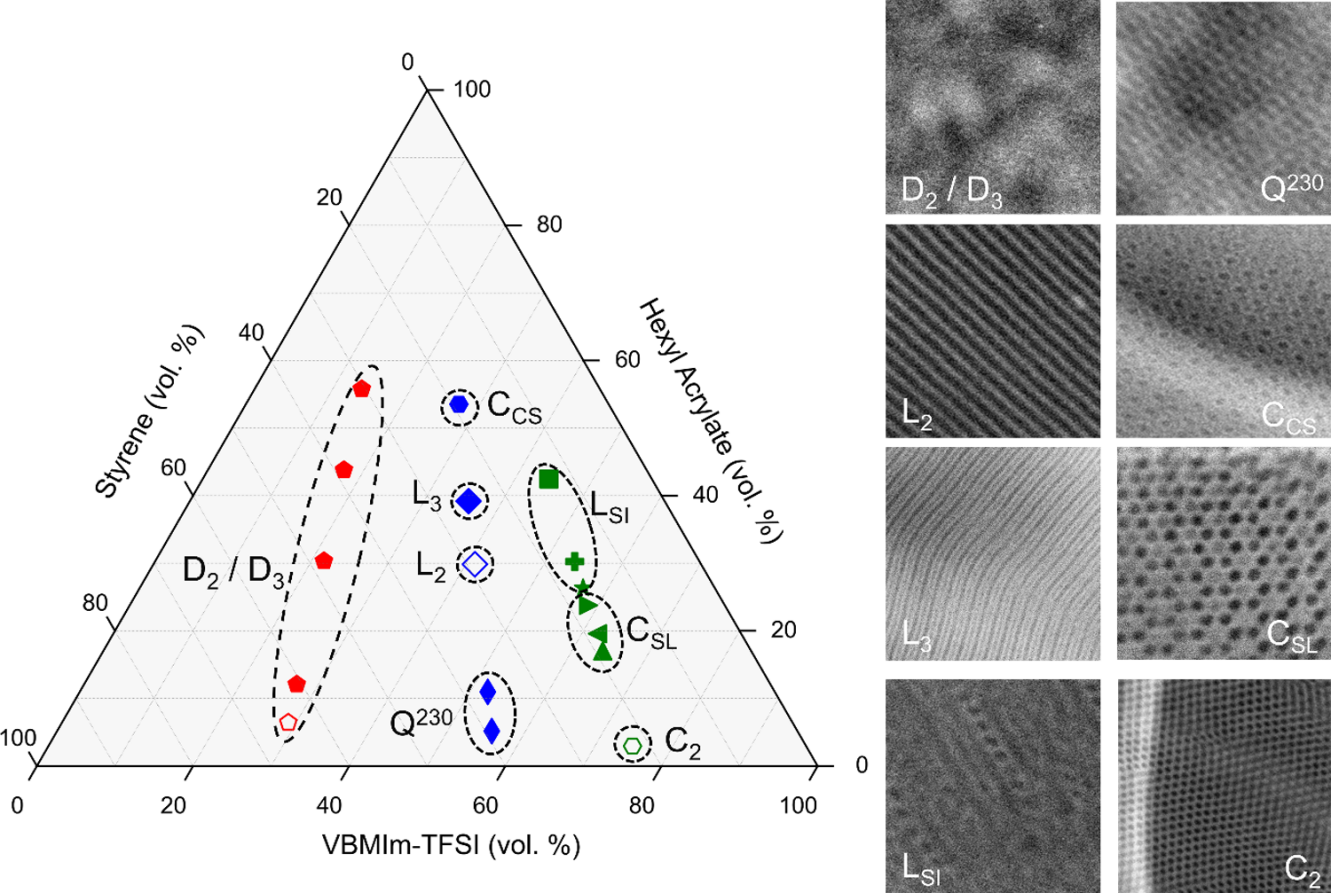
Morphology phase diagram of PIL ABC triblock terpolymer phase space: D_3_ – three-phase disordered microphase separation (filled red pentagon), D_2_ – two-phase disordered microphase separation (open red pentagon), C_CS_ – core–shell hexagonally packed cylinders (blue filled hexagon), L_3_ – three-domain ABCB lamellae (filled blue diamond), L_2_ – two-domain lamellae (open blue diamond), Q^230^ – core–shell double gyroid (blue filled thin diamond), L_SI_ – spheres in lamellae (green filled square, cross, and star), C_SL_ – hexagonal superlattice (green filled triangles), and C_2_ – two-phase hexagonally packed cylinders (green open hexagon).

All poly(S-*b*-VBMIm-TFSI-*b*-HA) compositions and their assigned morphologies are summarized in a ternary phase diagram (Figure 13). The 17 polymers are located in the region of the phase diagram bounded by 13% < ϕ_S_ < 65%, 14% < ϕ_PIL_ < 75%, and 3% < ϕ_HA_ < 56%. The polymers from series 1, 2, and 3 remain colored red, blue, and green, respectively. Open symbols represent a two-phase morphology, while filled symbols indicate a three-phase morphology. Pentagon symbols represent disordered morphologies (D_2_ and D_3_), thin diamonds represent the Q^230^ gyroid morphology, diamonds represent lamellar morphologies (L_2_ and L_3_), and triangles (C_SL_) and hexagons (C_2_ and C_CS_) represent cylindrical morphologies. The exceptions to this pattern are the samples that show the spheres-in-lamellae morphology (L_SI_), which are indicated by various symbol types (square, cross, and star).

In this view, the competing effects of strong immiscibility of the midblock (frustration), increasing segregation regime when moving from series 1 (red) to series 3 (green), and the effects of dispersity are more visible. As listed in Table 1, the polymers in series 1 all have 46 repeat units of S, 6 repeat units of VBMIm-TFSI, and increasing amounts of HA from 3 repeat units (46–6–3) to 55 repeat units (46–6–55), covering a broad region of the left side of the phase diagram (30% < ϕ_S_ < 65%, 14 < ϕ_VBMIm-TFSI_ < 30%, 5% < ϕ_HA_ < 56%). All the series 1 polymers have low total molecular weights and are likely not entangled. Regardless, all five samples (red pentagons in Figure 13) are microphase-separated. 46–6–3 shows scattering indicative of a two-phase microphase separated morphology lacking organization (D_2_), but presents strong structure factor scattering suggesting nondilute domains of VBMIm-TFSI in a matrix of S.^89^ This feature shifts paradoxically to higher *q* with increasing HA content, as indicated by the dashed line in Figure 5. One would naturally expect the addition of non-VBMIm-TFSI volume would drive the VBMIm-TFSI domains further apart, shifting this peak to lower *q*. Chen and co-workers^9^ observed this effect in a series of ABCBA pentablock terpolymers containing a PIL midblock (C) when swollen with IL. In that study, microphase separated domains of S assembled on a BCC lattice. The shift of the primary Bragg diffraction maximum to higher *q* with increasing IL content was attributed to a combination of reduced distance between S domains but also a reduction in average S domain size. Thus, the shift to higher *q* of the PIL structure factor peak may be due to a reduction in domain size along with reduced interdomain spacings. The remaining samples in series 1 also include either one or two lower-intensity peaks at lower angles than the peak associated with VBMIm-TFSI. From the discussion above and previous analyses in the literature,^52,54,74,75,77^ it is now understood that such features can be seen when a three-phase morphology is formed, which would indicate that in these samples HA is microphase separating with increasing HA content. The lack of structure observed by SAXS and TEM indicates that these samples have a disordered three-phase morphology (D_3_) and are therefore indicated by filled red pentagons in Figure 13. Combined, these observations confirm that the midblock, VBMIm-TFSI, is strongly immiscible in both end-blocks.

At the central region of the phase diagram, the morphological behavior of the polymers in series 2 (blue data in Figure 13) is more complex, due to both higher midblock content and higher molecular weight across the series. Compared to polymers in series 1, the VBMIm-TFSI content is increased from 6 to 19 repeat units and appears to place these 5 samples at least in the weak or even intermediate segregation regime. The lowest HA content samples, 46–19–4 and 46–19–9, both form the Q^230^ gyroid morphology, revealing a composition window which yields a 3D tri-co-continuous, triply periodic morphology (35% < ϕ_S_ < 40%, 50% < ϕ_VBMIm-TFSI_ < 60%, and 5% < ϕ_HA_ < 12%).

With increasing HA content, 46–19–31 (ϕ_S_ = 29%, ϕ_VBMIm-TFSI_ = 41%, and ϕ_HA_ = 30%) and 46–19–47 (ϕ_S_ = 25%, ϕ_VBMIm-TFSI_ = 36%, and ϕ_HA_ = 39%) both form lamellar morphology, locating near the center of the morphology phase diagram (where the volume percent composition of all blocks is close to 1/3 of the volume). The latter forms a three-phase ABCB-type lamellar structure, identical to that observed in some previous studies of ABC triblock terpolymers.^41,42,45,46,73−75^ Here, the HAADF-STEM data in Figure 7 clarify the morphological structure indicated by SAXS, which indicates the superposition of two different lamellar periods (13.0 and 26.1 nm). The lower X-ray contrast between S and VBMIm-TFSI + HA (or HA and S + VBMIm-TFSI) gives rise to the lower intensity primary Bragg diffraction maximum for the S/HA period, at *q* = 0.0241 Å^–1^. Surprisingly, a change in composition from 47 HA repeat units to 31 HA repeat units is enough to change the morphology from L_3_ to L_2_. This transition suggests that HA is more miscible than S in VBMIm-TFSI, consistent with a frustrated ABC triblock in which χ_PIL-S_ ≥ χ_PIL-HA_ ≥ χ_S-HA_, or χ_PIL-HA_ ≥ χ_PIL-S_ ≥ χ_S-HA_. The final polymer in this series, 46–19–84 (ϕ_S_ = 19%, ϕ_VBMIm-TFSI_ = 27%, and ϕ_HA_ = 54%), comprises high HA content in which HA serves as the matrix phase (Figure 8). The S end-block and VBMIm-TFSI midblock form cylinders in the HA, and the strong immiscibility of S in the VBMIm-TFSI block leads to the formation of a core–shell cylinder morphology with S surrounded by VBMIm-TFSI.

The third series of polymers, indicated by the green symbols in Figure 13, was found to have very interesting morphologies, some of which are only possible in ABC triblock terpolymers. This series has 46 repeat units of S and 45 repeat units of VBMIm-TFSI, more than double the PIL content of series 2, resulting in polymers with relatively high molecular weights. However, the dispersity of this series was also generally higher than the other two series, complicating the morphological formation. For 46–45–4 (ϕ_HA_ = 3%), the very low HA volume fraction leads to two dominant blocks (S and VBMIm-TFSI), and therefore the formation of a two-phase morphology of S cylinders in a VBMIm-TFSI matrix is expected (Figure 8). As HA content increases, the next three polymers in the series (46–45–27, 46–45–32, and 46–45–41) fall close together on the phase diagram in Figure 13. The SAXS and TEM data for these polymers shows the formation of superlattice of S and HA cylinders in a matrix of VBMIm-TFSI (filled green triangles in Figure 13), with a composition window at 17% < ϕ_S_ < 19%, 59% < ϕ_VBMIm-TFSI_ < 64%, 17% < ϕ_HA_ < 24%. This morphology has been reported previously in nonionic but highly frustrated ABC triblock terpolymers.^77,90^ These reports of superimposed hexagonal lattices are distinct from findings of tetragonal superlattices, or hexagonal lattices with cylinders in linear arrays, for nonfrustrated triblock terpolymers,^45,47^ and confirm the large enthalpic driving force for VBMIm-TFSI to separate from both end-blocks simultaneously.

The most intriguing morphology observed for the ABC PIL triblock terpolymers is the spheres-in-lamellae (L_SI_) structure shown in Figures 11 and 12. Samples 46–45–47 and 46–45–57 (solid green star and cross in Figure 13) exhibit the L_SI_ phase, which locates adjacent to the C_SL_ window at 15% < ϕ_S_ < 18%, 54% < ϕ_VBMIm-TFSI_ < 57%, and 25% < ϕ_HA_ < 30%. This morphology was reported by Shibayama et al. in 1982,^91^ for a sample of poly(styrene-*b*-(4-vinylbenzyl)dimethylamine-*b*-isoprene), but their microscopy data do not show any images representing the view from arrow 1 in Figure 11, and their SAXS data do not show the combination of lamellar and hexagonal order observed in this study. These differences, and the lack of any subsequent reports of this morphology,^39^ suggest that the behavior observed for 46–45–47 and 46–45–57 is new. Here, the VBMIm-TFSI and HA form a lamellar morphology, and the S end-blocks microphase separate into spheres within the VBMIm-TFSI domains. It is important to note that this morphology is different from that reported by Löbling et al.,^52^ of cylinders in lamellae found for poly(styrene-*b*-butadiene-*b*-*tert*-butyl methacrylate), another strongly frustrated system. In that morphology, the cylinders lie in the plane of the lamellae, resulting in a view of the hexagonal ordering of the cylinders only when the lamellae are viewed in the plane of the lamellae (views 2 and 3 in Figure 11). In samples studied here, the hexagonal lattice is only visible when viewed normal to the plane of the lamellae. The observation of spheres-in-lamellae is consistent with the segmental interaction parameters placing the ABC triblock terpolymers in the highest frustration category.

The last sample in series 3, 46–45–97, has a morphology that appears also to have lamellar and spherical character, but is unable to be determined from TEM or SAXS. The TEM data offer compelling support for including this morphology in the spheres-in-lamellae category, with notable similarities to the images in Figure 11. It is also noteworthy that the dispersity of this sample is the highest of all samples studied in this work, 1.88. It remains unclear whether the midblock is less miscible in PS or PHA, but in either case, it is safe to conclude that both χ_PIL-HA_ and χ_PIL-S_ are significantly greater than χ_S-HA_.

### Ion Conductivity

Multiple studies have investigated ion transport in PIL AB diblock copolymers and revealed that the conductivity is strongly related to the morphology, which is impacted by ionic block content, *T*_g_, and the film processing conditions.^19,92^ Note that, in this study, the *T*_g_s of the conducting block are constant at ca. 30 °C and the film processing conditions were kept the same, resulting in minimal impact on changing morphology and subsequently the ion conductivity of the polymers. Therefore, changing composition is the only parameter that impacts morphology in this study, which allows for an exclusive systematic study of conductivity–morphology relationships in PIL ABC triblock terpolymers.

**Figure 14:**
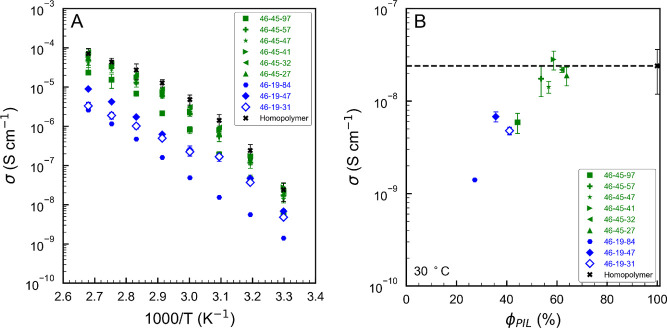
Ion conductivity of poly(S-*b*-VBMIm-TFSI-*b*-HA) polymers as a function of (A) temperature and (B) conducting block volume fraction.

The through-plane ionic conductivity of select PIL ABC triblock terpolymers was measured over a range of temperatures (30 to 100 °C). The conductivity for select polymers (i.e., 46–6–3, 46–6–6, 46–6–19, 46–6–34, 46–6–55, 46–19–4, 46–19–9, 46–45–6) were not measurable due to the insufficient mechanical properties of the polymer films. Figure 14A shows the temperature-dependent ion conductivity under a dry condition for the PIL ABC triblock terpolymers with a variety of PIL block compositions and the PIL homopolymer, poly(VBMIm-TFSI). Conductivity values at 30 °C were listed in Table 3. The conductivity increases over 3 orders of magnitude with increasing temperature from 30 to 100 °C and reaches the maximum conductivity at 100 °C for all polymers. 46–45–32 achieved the highest conductivity of 7.71 × 10^–5^ S cm^–1^ among the PIL ABC triblock terpolymers at 100 °C. Surprisingly, the PIL ABC triblock terpolymers with higher PIL block composition (54% < ϕ_VBMIm-TFSI_ < 64%) show similar conductivity compared to their analogous homopolymer across the temperature range (e.g., 2.17 × 10^–8^ S cm^–1^ for 46–45–32 versus 2.40 × 10^–8^ S cm^–1^ for homopolymer at 30 °C, and 7.71 × 10^–5^ S cm^–1^ for 46–45–32 versus 7.07 × 10^–5^ S cm^–1^ for homopolymer at 100 °C) with lower conducting block volume fraction (e.g., 62% for 46–45–32 versus 100% for poly(VBMIm-TFSI)), indicating that the ionic conductivity is not solely dependent on the PIL composition.

**Table 3:** Volume Fraction, Morphology, Conductivity, and Morphology Factor of Select Poly(S-*b*-VBMIm-TFSI-*b*-HA)

polymer (*n*-*m*-*p*)^a^	ϕ_s_^b^ (%)	ϕ_PIL_^b^ (%)	ϕ_HA_^b^ (%)	morphology	σ^c^ (1 × 10^–9^ S cm^–1^)	morphology factor *f*^d^
46–19–31	29	41	30	L_2_	4.76	0.48
46–19–47	25	36	39	L_3_	6.79	0.79
46–19–84	19	27	54	C_CS_	1.40	0.21
46–45–27	19	64	17	C_SL_	18.9	1.23
46–45–32	18	62	20	C_SL_	21.7	1.46
46–45–41	17	59	24	C_SL_	28.1	2.00
46–45–47	17	57	26	L_SI_	14.2	1.04
46–45–57	16	54	30	L_SI_	17.4	1.35
46–45–97	13	44	42	L_SI_*	5.88	0.55

a Numbers (*n*, *m*, and *p*) represent the number of repeat units in the S, VBMIm-TFSI, and HA blocks, respectively, as determined by ^1^H NMR spectroscopy.

b Volume fractions calculated from density of polystyrene (1.05 g cm^–3^), poly(VBMIm-TFSI) (1.41 g cm^–3^), and poly(hexyl acrylate) (1.05 g cm^–3^) (volume fraction calculation can be found in Supporting Information).

c Conductivity measured at 30 °C.

d Morphology factors at 30 °C calculated from eq 2.

Figure 14B shows the ionic conductivity of PIL ABC triblock terpolymers and PIL homopolymer as a function of PIL volume fraction at 30 °C. Polymers with higher PIL block composition (54% < ϕ_VBMIm-TFSI_ < 64%) show ca. an order of magnitude higher conductivity than polymers with lower conducting block compositions (27% < ϕ_VBMIm-TFSI_ < 45%). It is clear that the ion conductivity increases significantly with increasing PIL composition, however, in a nonlinear fashion. For example, 46–19–84 shows the lowest conductivity (1.40 × 10^–9^ S cm^–1^ at 30 °C) among the triblock terpolymers with a PIL volume fraction of 27%. When the PIL volume fraction increases from 27% (46–19–84) to 36% (46–19–47), the conductivity increases by nearly 5-fold from 1.40 × 10^–9^ to 6.79 × 10^–9^ S cm^–1^. With continuous increase of the PIL volume fraction, the conductivity increases by over 1 order of magnitude (30-fold increase, from 1.40 × 10^–9^ S cm^–1^ for 46–19–84 to 2.81 × 10^–8^ S cm^–1^ for 46–45–41), while the PIL block volume fraction changed by only ca. 2-fold. With only small changes in the conducting volume (a minimum of 27% to a maximum of 64% in this series of polymers), substantial increase in conductivity and morphology factors (described below) were observed. These results indicate that the change of morphology types has a significant impact on the ion conduction of the polymers. In addition, it is more prominent in Figure 14B that the PIL ABC triblock terpolymers achieved similar ion conductivity as their analogous homopolymer (dashed line) with lower ion density, suggesting that ion conduction is accelerated for certain nanostructured morphologies.

**Figure 15:**
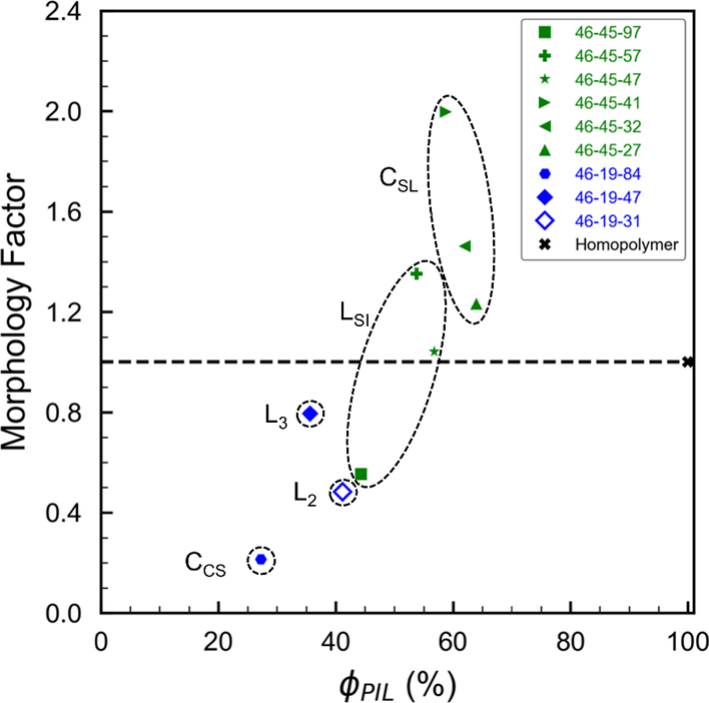
Morphology factor versus conducting block volume fraction of poly(S-*b*-VBMIm-TFSI-*b*-HA) at 30 °C. PIL homopolymer (poly(VBMIm-TFSI)) with a morphology factor of 1.00 is indicated with a dashed line for comparison.

Previous studies have observed similar results where PIL block copolymers achieved similar or higher conductivity compared to their analogous homopolymers with higher PIL content, and the conductivity increases nonlinearly with respect to increasing PIL block composition.^10,93,94^ This phenomena was attributed to the microphase-separated nanoscale morphology of the block copolymers, which leads to accelerated ion transport. To investigate the morphology-conductivity correlations in block copolymers containing both conducting and nonconducting phases, several studies^19,92,94^ proposed the calculation of a morphology factor (f, normalized ion conductivity) with the following equation:
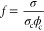
2where σ is the measured ionic conductivity of the block copolymer, σ_c_ and ϕ_c_ are the intrinsic ionic conductivity and volume fraction of the conducting microdomain, respectively. For a single-ion conducting PIL block polymer, the ion conductivity of the PIL homopolymer is used as σ_c_, and the volume fraction of PIL block is used as ϕ_c_. Figure 15 shows the morphology factor versus PIL volume fraction of a series of poly(S-*b*-VBMIm-TFSI-*b*-HA) under a dry condition at 30 °C. PIL homopolymer (poly(VBMIm-TFSI)) with a morphology factor of 1.00 is indicated with a dashed line for comparison. At the low PIL volume fractions (ϕ_PIL_ < 50%), 46–19–84 (ϕ_PIL_: 27%), with a C_CS_ morphology containing PIL as the shell of the cylinders, the polymer achieves the lowest morphology factor of 0.21 compared to the other polymers. For this polymer, the matrix is HA with a core of S cylinders surrounded by a shell of the conducting VBMIm-TFSI, where 1D transport pathway and the lack of adequate connectivity of these PIL shells probably leads to low morphology factor. The morphology factor is also comparable to the theoretical value of a randomly oriented 1D hexagonally packed cylindrical morphology (*f*_ideal,C_ = 1/3). When the conducting volume fraction continues to increase from 27% to 36% and 41%, the morphology factor increases significantly, which coincides with the morphology change from core–shell hexagonally packed cylinders to lamellae. The morphology factor for 46–19–47 (ϕ_PIL_: 36%) with L_3_ is 0.79 and the morphology factor for 46–19–31 (ϕ_PIL_: 41%) with L_2_ is 0.48, which are also comparable to the theoretical value of a randomly oriented 2D lamellar morphology (*f*_ideal,L_ = 2/3). The increase in the morphology factor can be attributed to the facilitated ion transport in the 2D ion-conducting pathways of lamellae compared to the 1D hexagonally packed cylinders.

At higher PIL volume fractions (ϕ_PIL_ > 50%), morphology factors of 1.35, 1.04, 2.00, 1.46, and 1.23 were observed for PIL triblock terpolymers with PIL volume fractions of 54, 57, 59, 62, and 64%, respectively. These unusually high morphology factors (i.e., exceeding the morphology factor of the homopolymer (1.00)) are consistent with the conductivity data, where five PIL triblock terpolymers with lower PIL volume fraction achieved similar conductivity compared to their homopolymer analogs (Figure 14B). Specifically, polymers with morphology factors of 2.00 (46–45–41; ϕ_PIL_: 59%), 1.46 (46–45–32; ϕ_PIL_: 62%) and 1.23 (46–45–27; ϕ_PIL_: 64%) exhibit C_SL_ morphology as evidenced by the SAXS profiles and TEM images (Figure 9). Note that, in these polymers, the C_SL_ morphology displays a superlattice with an overall 2D hexagonal array of cylinders (S and HA) embedded in a 3D continuous matrix of the PIL, which is the conducting phase. To understand the increase in the morphology factor of polymers with the C_SL_ morphology, we calculated the size of the ion-conducting PIL domain based on the domain spacing and lattice parameter obtained from SAXS, as well as the geometric relationship of the unit cell and the volume fraction of each block. The details of the calculations were explained in the Supporting Information (Section S5, Figure S6A). The conducting PIL phase between the S and HA cylinders appears to be ca. 6–10 nm, suggesting the formation of highly ordered ion-conducting nanochannels. The exceptionally high morphology factors can be attributed to these well-connected 3D continuous narrow ion transport pathways, which increase the local ion concentration and accelerate the ion conduction. This nanoscale confinement effect has previously been highlighted by Park,^95^ where enhanced conductivity was observed in block copolymer electrolytes by confining ions in narrow nanodomains.^96,97^

At slightly lower PIL volume fraction, polymers with morphology factors of 1.35 (46–45–57; ϕ_PIL_: 54%) and 1.04 (46–45–47; ϕ_PIL_: 57%) exhibit L_SI_ morphology as evidenced by SAXS and TEM results (Figure 11). L_SI_ morphology consists of alternating HA and PIL lamellae, while the S block microphase separates into spheres within the VBMIm-TFSI lamellae. The conducting PIL phase between the S spheres appears to be ca. 5–8 nm for 46–45–57 (Section S5, Figure S6B), again suggesting the formation of highly ordered ion conducting nanochannels. Interestingly, the L_SI_ morphology factors for are higher than the L_3_ and L_2_ morphologies and the theoretical value of a randomly oriented 2D lamellar morphology (*f*_ideal, L_ = 2/3). This may be attributed to narrow (ca. 5–8 nm) conducting PIL phase between the S spheres within in the lamellae, which may accelerate ion transport compared to the analogous randomly oriented 2D lamellar morphology. One exception is that 46–45–97 (ϕ_PIL_: 44%) obtained a morphology factor of 0.55, which is significantly lower than the other polymers with the L_SI_ morphology. This could be attributed to the mixed morphology (i.e., majority of microphase separated, but disordered structure with parts of sphere-in-lamellae morphology and lamellae morphology) and large dispersity of the polymers, which results in the discontinuity of the conducting phase.

Overall, PIL ABC triblock terpolymers with nanostructured network morphology achieve exceptionally high morphology factor exceeding their homopolymer analogs, suggesting the significant enhancement effect of morphology on the ion transport. This study provides valuable insights on the morphology–conductivity correlation in single-ion conducting ABC triblock terpolymers. Future work will focus on investigating the ion conduction mechanism in morphology with high morphology factors, i.e., expanding the composition region on the ternary phase diagram to establish a deeper understanding on the phase behavior of highly frustrated ion conducting PIL ABC triblock terpolymer systems.

## Conclusions

In conclusion, 17 compositions of a highly frustrated PIL ABC triblock terpolymer, poly(S-*b*-VBMIm-TFSI-*b*-HA) (S = styrene, VBMIm-TFSI = vinylbenzyl methylimidazolium bis(trifluoromethylsulfonyl)imide, HA = hexyl acrylate), were synthesized in this study via RAFT polymerization followed by postpolymerization functionalization and ion exchange reactions. This allowed for a systematic study of the impact of block composition on morphology and ion conductivity. A ternary morphology phase diagram was constructed, where nine morphologies including triply periodic Q^230^ gyroid, core–shell hexagonally packed cylinders, hexagonal superlattice, two-phase and three-phase lamellae, and sphere-in-lamellae were observed at different polymer compositions evidenced by SAXS and HAADF-STEM. A 3D tri-co-continuous, triply periodic Q^230^ morphology composition window was observed at 51% < ϕ_VBMIm–TFSI_ < 57%, 4% < ϕ_HA_ < 12%, and 36% < ϕ_S_ < 40%. An exceptionally high morphology factor (i.e., normalized ion conductivity) of 2.0 was observed by the PIL ABC triblock terpolymer with a hexagonal superlattice morphology, attributed to the formation of 3D narrow continuous PIL nanodomains that lead to accelerated ion conduction. Remarkable change in morphology factors were revealed with only a small change in the conducting volume due to the change of morphology types, indicating a significant impact of morphology on accelerating the ion conduction. Overall, this work demonstrates, for the first time, highly frustrated PIL ABC triblock terpolymers with nine nanostructured morphologies and morphology–conductivity correlations, where the hexagonal superlattice morphology with 3D continuous narrow ion-conducting channels achieves exceptionally high morphology factors.

## Supporting Information Available

Synthesis and chemical characterization of poly(ionic liquid) (PIL) ABC triblock terpolymer; density estimation of poly(VBMIm-TFSI), volume composition calculations of poly(S-*b*-VBMIm-TFSI-*b*-HA), morphology of block polymers, and calculation of the size of ion transport channels (PDF)
