# Supplementary material for: Highly Frustrated Poly(ionic liquid) ABC Triblock Terpolymers with Exceptionally High Morphology Factors
Source: Macromolecules. 2024 Apr 2;57(8):3776–97. doi: 10.1021/acs.macromol.3c02435 (PMC11044597; doi:10.1021/acs.macromol.3c02435)
Supplement: Supplementary file 1 — ma3c02435_si_001.pdf [file ma3c02435_si_001.pdf]

Supporting Information

Highly Frustrated Poly(ionic liquid) ABC Triblock Terpolymers with Exceptionally High Morphology Factors

Patrick M. Lathrop,[1,+] *Rui Sun,*[1,+] *Frederick L. Beyer,*[2]* *and Yossef A. Elabd,*[1]*

[1]Department of Chemical Engineering, Texas A&M University, College Station, TX 77843

[2]U.S. Army Research Laboratory, Aberdeen Proving Ground, MD, 21005

* To whom correspondence should be addressed; E-mail: elabd@tamu.edu, frederick.l.beyer3.civ@army.mil

[+] Patrick M. Lathrop and Rui Sun contribute equally to this work

S1. Synthesis and Chemical Characterization of Poly(ionic liquid) (PIL) ABC Triblock Terpolymer.

Table S1. Polymer names, structures, and labeling convention.

Polymer	Polymer Structure[a]	Polymer Composition Label[b]
PS macro-CTA	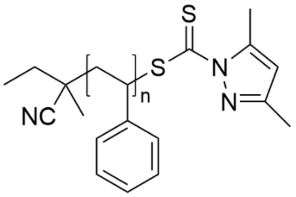	n
Poly(S-*b*-VBC)	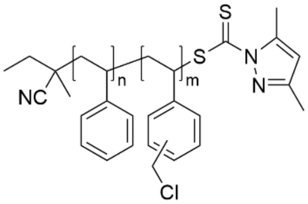	n-m
Poly(S-*b*-VBC-*b*-HA)	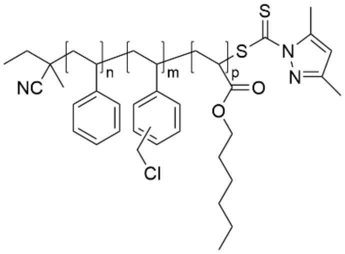	n-m-p-neutral
Poly(S-*b*-VBMIm-Cl-*b*-HA)	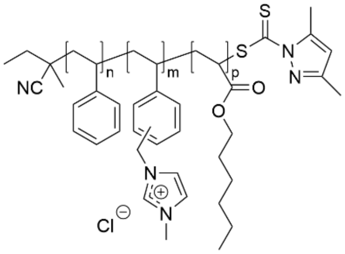	n-m-p-Cl
Poly(S-*b*-VBMIm-TFSI-*b*-HA)	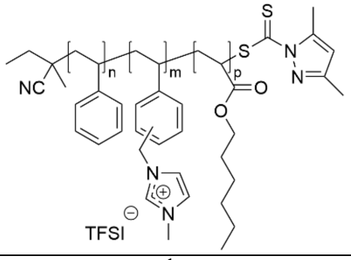	n-m-p

[a]Synthesis of each structure is shown in Scheme 1; [b]n, m, and p represent the number of repeat units in the first, second, and third block, respectively, of poly(S-*b*-VBMIm-TFSI-*b*-HA), as determined by ^{1}H nuclear magnetic resonance (NMR) spectroscopy; neutral represent the neutral ABC triblock terpolymer precursor of poly(S-*b*-VBC-*b*-HA), and Cl represent the mobile counterion on the PIL blocks in poly(S-*b*-VBMIm-Cl-*b*-HA).

Table S2. Reaction conditions for RAFT polymerizations of neutral ABC triblock terpolymers.

Product Composition Label[a]	Precursor	Recipe[b]	Degas Time (h)	Reaction Time (h)
46	CTA	100:1:0	2	20
46-6	46	65:1:0.2	1	1
46-19	46	100:1:0.2	1	3
46-45	46	100:1:0.2	1	8
46-6-3-neutral	46-6	100:1:0.1	0.5	0.5
46-6-6-neutral	46-6	100:1:0.1	0.5	0.75
46-6-19-neutral	46-6	100:1:0.1	0.5	1
46-6-34-neutral	46-6	100:1:0.1	0.5	2
46-6-55-neutral	46-6	100:1:0.1	0.5	3
46-19-4-neutral	46-19	100:1:0.1	0.5	0.5
46-19-9-neutral	46-19	100:1:0.1	0.5	1
46-19-31-neutral	46-19	100:1:0.1	0.5	2
46-19-47-neutral	46-19	100:1:0.1	0.5	3
46-19-84-neutral	46-19	100:1:0.1	0.5	4
46-45-4-neutral	46-45	100:1:0.1	0.5	1
46-45-27-neutral	46-45	100:1:0.1	0.5	1.5
46-41-32-neutral	46-45	100:1:0.1	0.5	2
46-45-41-neutral	46-45	100:1:0.1	0.5	3
46-45-47-neutral	46-45	100:1:0.1	0.5	4
46-45-57-neutral	46-45	100:1:0.1	0.5	5
46-45-97-neutral	46-45	150:1:0.1	0.5	8

[a]Numbers represent number of repeat units of each block as determined by ^{1}H NMR spectroscopy in the order given in the polymer name; [b]recipe shown as molar ratio (monomer:precursor:initiator); [c]monomer:solvent weight ratio.

Table S3. Product yields, molecular weights, dispersities, and thermal transitions of PIL ABC triblock terpolymers and their respective precursors.

Polymer Composition Label[a]	Yield (g)	Yield (%)	M_n[b] (Da)	M_w[b] (Da)	Đ[b]	$T_{g,1}$[c] (°C)	$T_{g,2}$[c] (°C)	$T_{g,3}$[c] (°C)	T_d[d] (°C)
46	331.03	97.6	5150	6184	1.20	92	-	-	322
46-6	93.81	88.2	5569	6614	1.19	96	-	-	305
46-19	125.03	88.2	7575	9134	1.21	93	-	-	310
46-45	139.04	84.6	8791	11251	1.36	98	-	-	313

46-6-3-neutral	15.74	91.1	4877	5797	1.19	71	-	-	291
46-6-6-neutral	12.85	74.0	5448	6559	1.20	62	-	-	292
46-6-19-neutral	13.85	61.6	6547	7943	1.21	37	-	-	301
46-6-34-neutral	18.62	65.6	7726	9978	1.29	-3	-	-	304
46-6-55-neutral	20.55	56.1	8441	11334	1.34	-35	-	-	300
46-19-4-neutral	18.95	87.9	5788	7060	1.22	61	-	-	285
46-19-9-neutral	17.51	74.4	6062	7448	1.23	64	-	-	290
46-19-31-neutral	12.58	39.1	9604	12478	1.30	25	-	-	306
46-19-47-neutral	19.49	50.6	10245	13582	1.33	-3	-	-	302
46-19-84-neutral	25.32	47.7	11660	17431	1.50	-51	-	-	303
46-45-4-neutral	6.42	30.5	8976	12241	1.36	81	-	-	297
46-45-27-neutral	16.55	61.1	12600	19310	1.53	61	-	-	322
46-41-32-neutral	26.89	80.6	12450	19050	1.53	56	-	-	313
46-45-41-neutral	22.32	72.6	12852	20322	1.58	58	-	-	318
46-45-47-neutral	25.44	78.7	13332	21934	1.65	65	-45	-	321
46-45-57-neutral	29.34	83.9	13508	22720	1.68	67	-49	-	313
46-45-97-neutral	30.76	90.3	14901	26914	1.81	70	-53	-	324
46-6-3-Cl	14.24	88.2	6921[e]	8229[f]	1.19	90	-	-	241
46-6-6-Cl	10.56	98.6	7390[e]	8898[f]	1.20	77	-	-	244
46-6-19-Cl	13.62	86.1	9421[e]	11428[f]	1.21	52	-	-	251
46-6-34-Cl	13.73	87.7	11764[e]	15187[f]	1.29	19	-	-	266
46-6-55-Cl	8.00	77.4	15045[e]	20205[f]	1.34	-40	-	-	254
46-19-4-Cl	8.29	70.1	10129[e]	12357[f]	1.22	157	100	-	242
46-19-9-Cl	11.52	98.7	10910[e]	13408[f]	1.23	153	95	-	237
46-19-31-Cl	2.88	25.7	14347[e]	18637[f]	1.30	143	26	-	241
46-19-47-Cl	3.89	35.3	16847[e]	22339[f]	1.33	143	-38	-	244
46-19-84-Cl	10.98	99.9	22627[e]	33827[f]	1.50	131	-52	-	249
46-45-4-Cl	4.75	73.4	16232[e]	22140[f]	1.36	174	93	-	230
46-45-27-Cl	8.74	71.1	19826[e]	30393[f]	1.53	176	-	-	239
46-41-32-Cl	9.15	50.1	20607[e]	31591[f]	1.53	174	-40	-	240
46-45-41-Cl	13.26	73.6	22013[e]	34803[f]	1.58	174	117	-52	242
46-45-47-Cl	9.34	52.2	22950[e]	37753[f]	1.65	176	108	-50	244
46-45-57-Cl	12.61	71.4	24512[e]	41229[f]	1.68	176	94	-51	243
46-45-97-Cl	6.48	38.0	30761[e]	55554[f]	1.81	175	118	-53	242
46-6-3	15.89	94.7	8389[e]	9975[f]	1.19	83	-	-	327
46-6-6	11.12	91.3	8858[e]	10665[f]	1.20	72	-	-	333
46-6-19	13.75	90.0	10889[e]	13208[f]	1.21	30	-	-	336
46-6-34	14.26	95.0	13252[e]	17108[f]	1.29	14	-	-	333
46-6-55	7.16	85.8	16513[e]	22177[f]	1.34	-40	-	-	324
46-19-4	10.96	95.3	14778[e]	18029[f]	1.22	84	29	-	348

46-19-9	13.56	85.5	15559[e]	19122[f]	1.23	78	30	-	341
46-19-31	3.01	91.6	18996[e]	24676[f]	1.30	93	24	-19	346
46-19-47	4.22	94.6	21496[e]	28504[f]	1.33	22	-40	-	345
46-19-84	12.04	90.5	27276[e]	40778[f]	1.50	21	-53	-	338
46-45-4	5.49	87.3	27243[e]	37159[f]	1.36	82	37	-	341
46-45-27	10.26	85.4	30836[e]	47272[f]	1.53	33	-	-	328
46-41-32	10.95	87.6	31617[e]	48469[f]	1.53	32	-	-	324
46-45-41	9.70	52.7	33023[e]	52209[f]	1.58	76	27	-	324
46-45-47	11.80	95.8	33960[e]	55864[f]	1.65	29	-50	-	321
46-45-57	14.01	83.3	35526[e]	59755[f]	1.68	102	30	-52	328
46-45-97	6.94	93.3	41771[e]	75438[f]	1.81	102	29	-55	324

[a]Numbers represent repeat units of each block, as determined by ^{1}H NMR; [b]determined by size exclusion chromatography (SEC); [c]determined by differential scanning calorimetry (DSC); [d]determined by thermogravimetric analysis (TGA); [e]theoretical value calculated based on compositions determined by ^{1}H NMR spectroscopy; [f]theoretical value calculated based on compositions determined by ^{1}H NMR spectroscopy and dispersities determined by poly(S-*b*-VBC-*b*-HA) SEC results.

Table S4. Elemental analysis (EA) results for poly(S-*b*-VBMIm-Cl-*b*-HA).

**Polymer**	**C**		**H**		**N**		**S**		**Cl**	
	**Calc. (%)**	**Det. (%)**	**Calc. (%)**	**Det. (%)**	**Calc. (%)**	**Det. (%)**	**Calc. (%)**	**Det. (%)**	**Calc. (%)**	**Det. (%)**
46-6-3-Cl	83.99	85.27	7.59	7.69	3.04	2.18	0.93	0.80	3.07	1.90
46-6-6-Cl	83.05	84.27	7.76	7.94	2.84	2.10	0.87	0.80	2.88	1.81
46-6-19-Cl	80.06	81.96	8.31	8.36	2.23	1.71	0.68	0.54	2.26	1.79
46-6-34-Cl	77.90	79.30	8.71	8.70	1.79	1.35	0.55	0.41	1.81	1.39
46-6-55-Cl	76.00	77.02	9.06	9.21	1.40	1.04	0.43	0.55	1.41	0.91
46-19-4-Cl	78.50	76.25	7.28	7.35	5.67	5.39	0.63	0.63	6.65	6.51
46-19-9-Cl	77.83	77.70	7.50	7.32	5.26	5.19	0.59	0.51	6.17	6.22
46-19-31-Cl	75.76	74.75	8.18	8.35	4.00	3.59	0.45	0.51	4.69	4.13
46-19-47-Cl	74.79	74.75	8.50	8.35	3.41	3.35	0.38	0.39	4.00	3.72
46-19-84-Cl	73.36	73.23	8.96	9.07	2.54	2.53	0.28	0.20	2.98	2.72
46-45-4-Cl	74.00	73.16	6.97	7.03	8.03	7.94	0.40	0.23	9.83	9.83
46-45-27-Cl	73.12	71.58	7.58	7.71	6.57	6.37	0.32	0.15	8.05	7.92
46-45-32-Cl	72.98	71.30	7.68	7.62	6.32	6.21	0.31	0.10	7.74	7.63
46-45-41-Cl	72.73	71.78	7.85	7.75	5.92	6.00	0.29	0.08	7.25	7.47
46-45-47-Cl	72.59	71.97	7.95	7.87	5.68	5.83	0.28	0.00	6.95	7.38
46-45-57-Cl	72.37	70.92	8.10	8.11	5.31	5.45	0.26	0.00	6.51	6.83
46-45-97-Cl	71.73	70.74	8.55	8.41	4.24	4.81	0.21	0.00	5.19	6.22

Table S5. EA results for poly(S-*b*-VBMIm-TFSI-*b*-HA).

Polymer	C		H		N		S		Cl		F	
	Calc. (%)	Det. (%)	Calc. (%)	Det. (%)	Calc. (%)	Det. (%)	Calc. (%)	Det. (%)	Calc. (%)	Det. (%)	Calc. (%)	Det. (%)
46-6-3	71.01	75.82	6.26	6.66	3.51	2.59	5.35	4.06	0.00	0.00	8.15	5.96
46-6-6	70.91	76.35	6.47	7.01	3.32	2.40	5.07	3.48	0.00	Trace	7.72	5.25
46-6-19	70.59	75.40	7.19	7.49	2.70	2.09	4.12	3.12	0.00	Trace	6.28	4.90
46-6-34	70.34	73.89	7.75	7.96	2.22	1.72	3.39	2.59	0.00	0.00	5.17	3.94
46-6-55	70.12	71.92	8.26	8.62	1.78	1.42	2.72	2.36	0.00	0.00	4.14	3.62
46-19-4	56.89	57.06	4.99	4.97	5.69	5.57	8.68	8.57	0.00	0.00	14.66	14.92
46-19-9	57.51	57.72	5.26	5.25	5.40	5.49	8.24	8.46	0.00	0.00	13.92	14.14
46-19-34	59.62	61.34	6.18	6.27	4.42	4.13	6.75	6.29	0.00	0.00	11.40	10.57
46-19-47	60.74	61.80	6.66	5.62	3.91	3.85	5.97	6.62	0.00	0.00	10.08	9.78
46-19-84	62.53	63.92	7.43	7.53	3.08	3.00	4.70	4.49	0.00	0.00	7.94	8.40
46-45-4	48.05	48.67	4.15	4.16	7.10	6.91	10.83	10.43	0.00	0.00	18.83	18.48
46-45-27	50.52	50.50	4.87	4.90	6.27	6.19	9.57	9.37	0.00	0.00	16.64	16.80
46-41-32	50.98	51.08	5.00	4.99	6.11	6.03	9.33	9.06	0.00	0.00	16.23	16.36
46-45-41	51.75	51.86	5.23	5.15	5.85	5.97	8.93	8.87	0.00	0.00	15.53	15.68
46-45-47	52.24	52.32	5.37	5.29	5.69	5.83	8.69	8.64	0.00	0.00	15.11	15.40
46-45-57	52.98	52.76	5.59	5.53	5.44	5.58	8.30	8.25	0.00	0.00	14.44	14.72
46-45-97	55.41	54.18	6.30	5.98	4.63	5.16	7.06	7.35	0.00	0.00	12.28	13.69

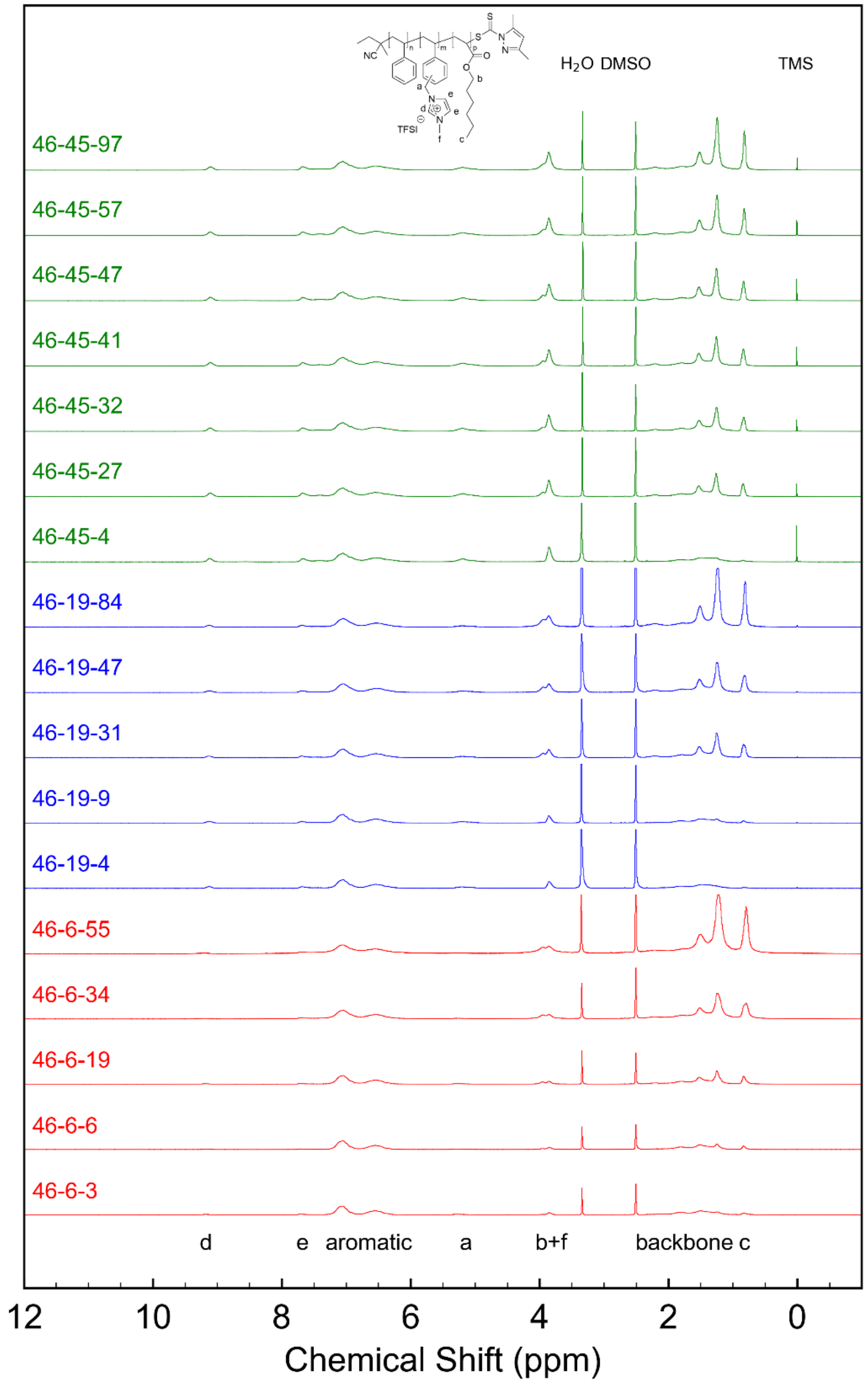

Figure S1. ^{1}H NMR spectra for poly(S-*b*-VBMIm-TFSI-*b*-HA).

Figure S2 shows all ^{19}F NMR spectra for all poly(S-*b*-VBMIm-TFSI-*b*-HA) polymers. Each spectrum shows one peak at -78.7 ppm which represents the fluorine atoms on the TFSI counter anion, indicating the presence of TFSI in the polymer and successful anion exchange reaction.

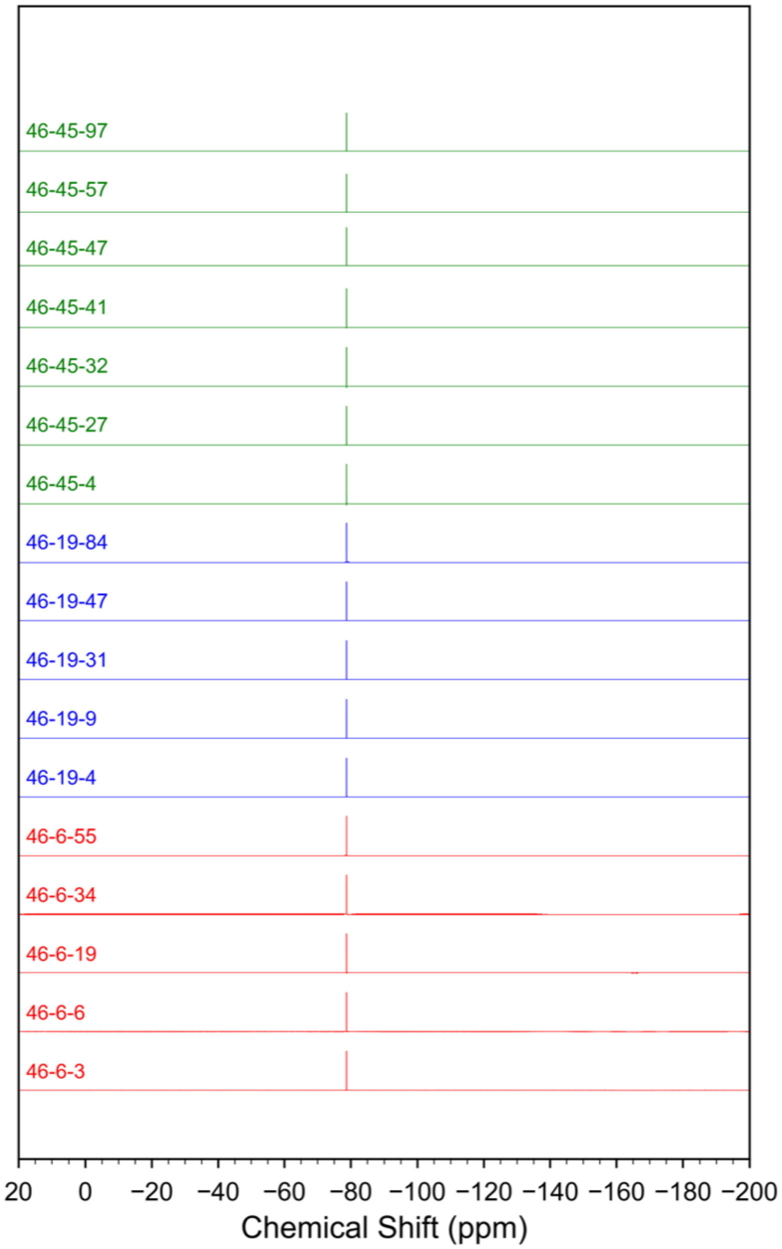

Figure S2. ^{19}F NMR spectra for poly(S-*b*-VBMIm-TFSI-*b*-HA).

The chemical structure of the polymers synthesized were further confirmed by ATR-FTIR spectroscopy (Figure S3). The spectra of the poly(S-*b*-VBMIm-TFSI-*b*-HA) and its precursors are shown in Figure S3 at one representative composition (46-19-47). The infrared band at 1260 cm^{-1} in Figure S3(II) and Figure S3(III) represents the C-Cl bond on the VBC block,[1] which confirms the successful chain-extension of the PS macro-CTA. The infrared bands at 1734, 1454, 1240 and 1167 cm^{-1} in Figure S3(III), Figure S3(IV), and Figure S3(V) are indicative of the C=O stretching,[2] C-O-C asymmetric stretching,[3] C-O stretching,[3] and C-(C=O)-O stretching[3] in the HA block, respectively, and show successful chain-extension of the poly(S-*b*-VBC). The appearance of the C-N stretching[4] infrared band at 1574 cm^{-1} (Figure S3(IV) and Figure S3(V)), and the absence of the infrared band for C-Cl at 1260 cm^{-1} (Figure S3(IV) and Figure S3(V)) suggest that both 46-19-47-Cl and 46-19-47-TFSI are functionalized.

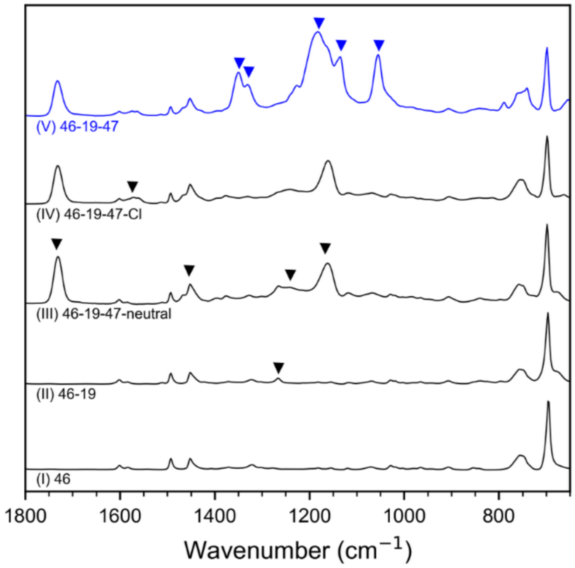

Figure S3. Representative ATR-FTIR spectra of (I) PS macro-CTA, (II) poly(S-*b*-VBC), (III) poly(S-*b*-VBC-*b*-HA), (IV) poly(S-*b*-VBMIm-Cl-*b*-HA), and (V) poly(S-*b*-VBMIm-TFSI-*b*-HA). Labels in inset correspond to the number of repeat units of each block and counter anion.

S2. Density Estimation of Poly(VBMIm-TFSI)

The density of the poly(VBMIm-TFSI) was estimated using an additive contribution technique.[5] Two components were used to represent poly(VBMIm-TFSI): poly(styrene) (PS) and ionic liquid (IL) 1,3-dimethylimidazolium bis((trifluoromethyl)sulfonyl)imide (Figure S4).

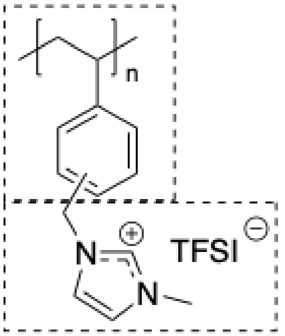

Figure S4. Chemical structure of poly(VBMIm-TFSI).

The density of the PIL block was calculated using Equation S1

$$\frac{1}{\rho_{PIL}} = \frac{{MW_{PS}}/{(MW_{PS}+MW_{IL})}}{\rho_{PS}} + \frac{{MW_{IL}}/{(MW_{PS}+MW_{IL})}}{\rho_{IL}} \qquad \text{Equation S1}$$

In equation S1, ρ_{PIL}, ρ_{PS}, and ρ_{IL} are the densities (g cm^{-3}) of poly(VBMIm-TFSI), polystyrene, and IL respectively. MW_{PIL}, MW_{PS}, and MW_{IL} are the molecular weights (g mol^{-1}) of the repeat units for poly(VBMIm-TFSI), PS, and IL respectively. The values used for calculation and the calculation results are summarized in Table S6.

Table S6. Molecular weight and density values for density calculation of poly(VBMIm-TFSI)

	PS	IL	PIL

MW (g/mol)	103.15[a]	376.27[a]	479.42
Density (g/cm^3)	1.04[b]	1.554[c]	1.405

[a]1 g mol^{-1} subtracted from the corresponding literature value (104.15 g mol^{-1} for S and 377.27 g mol^{-1} for IL, respectively) to account for the missing hydrogen atom due to the covalent bond between the two components. [b]Obtained from literature.[6] [c]Estimated from the group additivity method presented by Ye and Shreeve.[7]

S3. Volume Composition Calculations of Poly(S-*b*-VBMIm-TFSI-*b*-HA)

The volume percent composition of each block, i, in the poly(S-*b*-VBMIm-TFSI-*b*-HA) triblock terpolymers are calculated using Equation S2.

$$\phi_i = 100 \cdot x_i \frac{MW_i}{MW_p} \frac{\rho_p}{\rho_i} \qquad \text{Equation S2}$$

In Equation S2, x_i is the mole fraction composition of the ith block determined from the theoretical molecular weight, MW_i is the molecular weight of the ith block monomeric unit, MW_p is the average molecular weight of the repeat unit of the triblock terpolymer, ρ_i is the density of the ith block, and ρ_p is the density of the polymer (calculated by Equation S3).

$$\frac{1}{\rho_p} = \sum_{i=1}^{n} \frac{w_i}{\rho_i} \qquad \text{Equation S3}$$

where w_i is the weight fraction of the ith block. The values used for these Equation S2 and S3 are summarized in Table S7.

Table S7. Values used to calculate volume percent compositions of poly(S-*b*-VBMIm-TFSI-*b*-HA) polymers.

Property	Poly(styrene)	Poly(VBMIm-TFSI)	Poly(hexyl acrylate)
MW_i (Da)	104.15	479.42	156.22
ρ_i (g cm^{-3})	1.04	1.405	1.05

S4. Morphology of Block Polymers.

Table S8**.** Expected Bragg diffraction peak locations for common morphologies of block polymers.

**Morphology**[a]	**Expected Bragg Peak Locations**[b]	**Reference**
BCCS	1 √2, √3, 2	8
C	1, √3, 2, √7, 3, √12	8
L	1, 2, 3, 4, 5	9
G	1, √4/3, √8/3, √11/3	10
Q^{230}	√6, √8, √14, √16, √20, √22, √24, √26, √30, √32, √34, √38, √40, √42, √46, √48, √50	9
Q^{214}	√2, √6, √8, √10, √12, √14, √16, √18, √20	9
C_{CS}	1, √3, √4, √7, √9, √12, √13	11
O^{70}	1, 1.50, 2.53, 3.06, 3.23, 3.72, 3.97, 4.15, 4.76, 5.26, 5.64, 6.04, 6.29, 7.09, 7.30, 8.39, 9.84	9

[a]Acronyms: BCCS – BCC spheres; C – hexagonally packed cylinders; L – lamellar; G – gyroid; C_{CS} – hexagonally packed core-shell cylinders. [b]Expected Bragg peak locations are represented as the multiple of q* (the primary peak position in the SAXS profile).

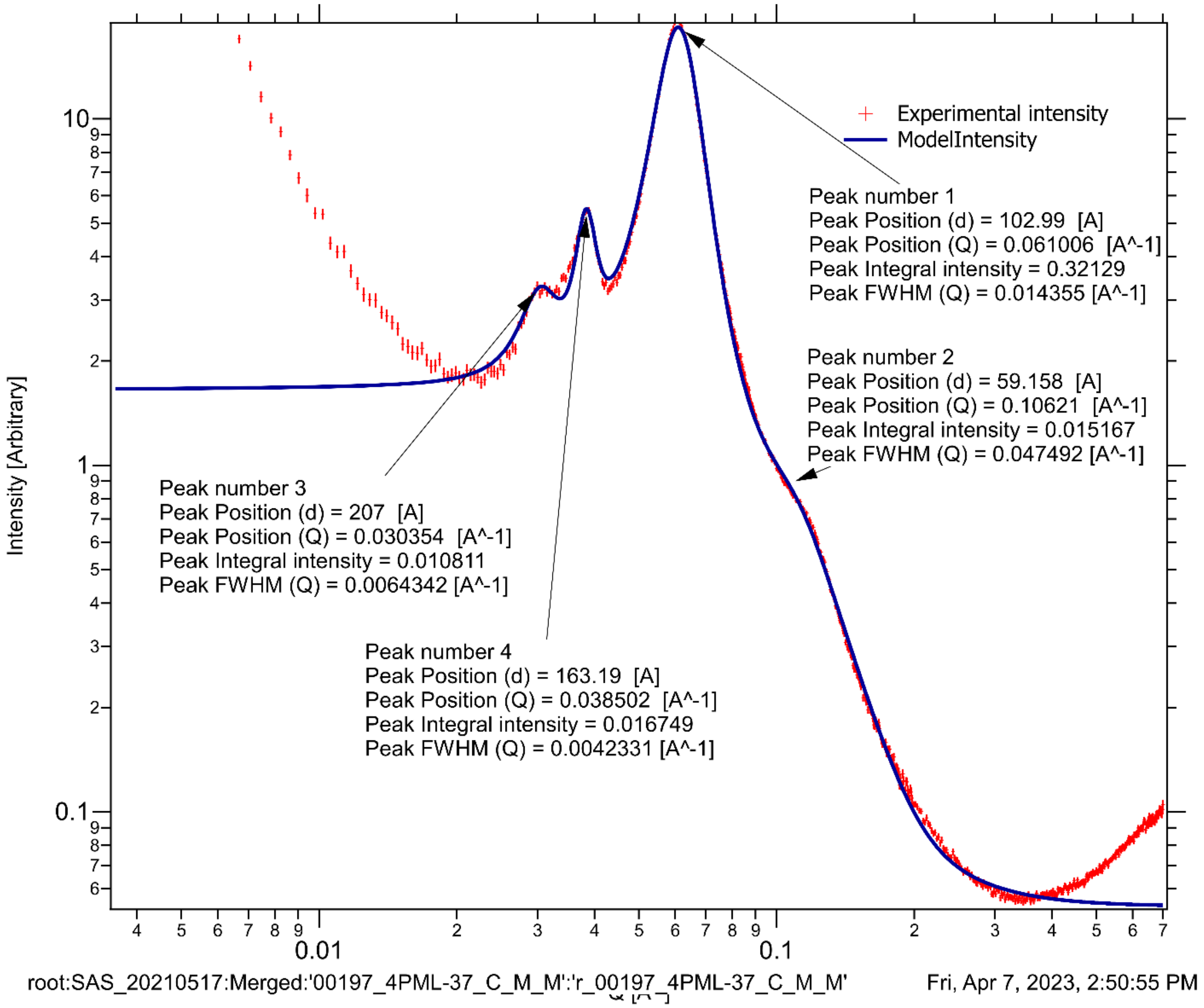

Figure S5. Typical peak fit for samples in the range 46-6-3 through 46-6-55, all of which produced SAXS data with a combination of weak and strong diffraction peaks. Here, for sample 46-6-6, the data have been fit with four Lorentzian distributions, in addition to a Guinier approximation and a constant background.

S5. Calculation of the Size of Ion Transport Channels

The calculation of the sizes of ion transport channels was performed based on the lattice parameter results from SAXS, the volume fraction of each block chemistry, and the geometric relations of the morphologies. Example calculations for C_{SL} morphology and L_{SI} morphology are shown below.

Schematic of a superlattice of hexagonally packed cylinders, viewed along the cylinder axis, is shown in Figure S6(A). The black trapezoid shows the primitive unit cell for the larger hexagonal structure, which contains the cross-section of two S cylinders, one HA cylinder, and PIL matrix. The area of each primitive unit cell for the larger hexagonal structure (A_{Large}) can be calculated using the lattice parameters for the large hexagonal lattices (a_{Large}) achieved from SAXS:

$$A_{Large} = \cos(30°) \times a_{Large}^2 \qquad \text{Equation S4}$$

The red trapezoid shows the primitive unit cell for the smaller hexagonal structure. The value of lattice parameter for the small hexagonal lattice (a_{Small}) could be obtained from both the SAXS data ($a_{Small,1}$) and geometry ($a_{Small,2}$), where

$$a_{Small,2} = \frac{a_{Large}}{2} \times \frac{2}{\sqrt{3}} = \frac{a_{Large}}{\sqrt{3}} \qquad \text{Equation S5}$$

The value we employed here is the average of the experimental and calculated values:

$$a_{Small} = \frac{a_{Small,1} + a_{Small,2}}{2} \qquad \text{Equation S6}$$

For cylindrical morphology, the area of each phase is proportional to the volume of each chemistry. Therefore, the radius of the cross-section of the S and HA cylinders (R_S and R_{HA}) can be calculated as:

$$R_S = \sqrt{\frac{A_S}{2\pi}} = \sqrt{\frac{A_{Large} \times \phi_S}{2\pi}} \qquad \text{Equation S7}$$

$$R_{HA} = \sqrt{\frac{A_{HA}}{\pi}} = \sqrt{\frac{A_{Large} \times \phi_{HA}}{\pi}} \qquad \text{Equation S8}$$

where A_S and A_{HA} are the cross-section areas of S cylinders and HA cylinders, respectively. ϕ_S and ϕ_{HA} are the volume fractions for S and HA blocks, respectively.

As a result, the size of ion transport channels in the C_{SL} morphology (d_1, d_2, and d_3 as indicated in Figure S6(A)) can be calculated based on the geometric relations:

$$d_1 = a_{Small} - R_S - R_{HA}$$ Equation S9

$$d_2 = a_{Small} - R_S - R_S$$ Equation S10

$$d_3 = a_{Large} - R_{HA} - R_{HA}$$ Equation S11

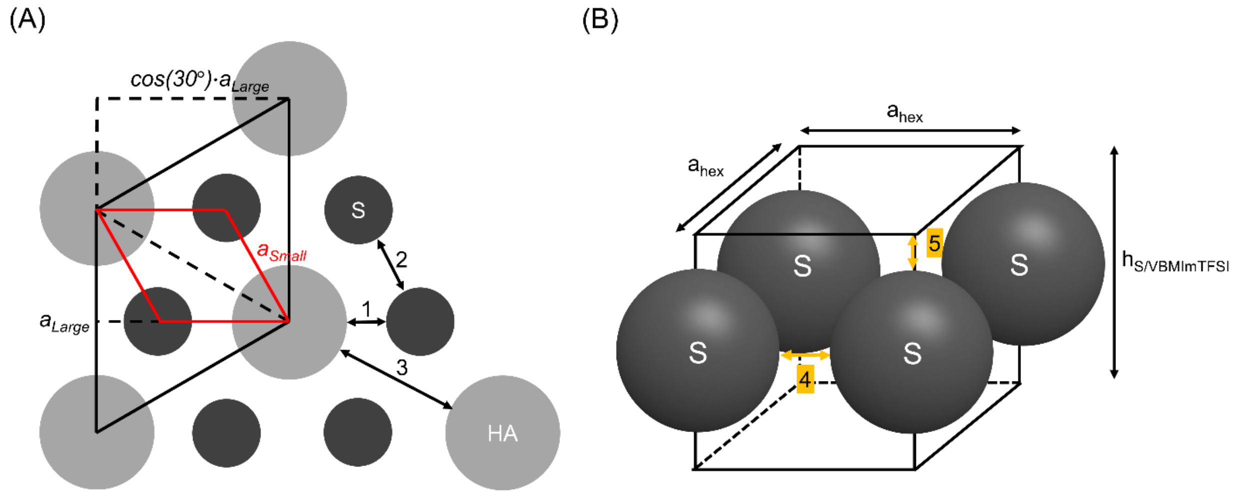

Figure S6. (A) Schematic of a superlattice of hexagonally packed cylinders, viewed along the cylinder axis. Domain shadings correspond to mean free path calculations (S is dark gray, HA is intermediate gray). (B) Schematic of a unit cell of the S spheres (dark gray) in PIL lamellae (white) in the L_{SI} morphology. The spacing for ion transport channels are marked as channel 1, 2, 3 in (A) and channels 4 and 5 in (B).

For the L_{SI} morphology, the schematic of a unit cell of the S spheres (dark gray) in PIL lamellae (white) is shown in Figure S6(B). The unit cell contains one S sphere and the rest is the PIL matrix. For lamellae morphology, the total thickness of the lamellar period (d), which are achieved from SAXS results, is proportional to the volume of each chemistry that consists the lamellae. Since the morphology consists of alternating HA lamellae and VBMImTFSI lamellae with spheres of S, the thickness of the S/VBMImTFSI lamellae layer ($h_{S/VBMImTFSI}$) can be calculated as:

$$h_{S/VBMImTFSI} = (\phi_S + \phi_{VBMImTFSI}) \times d$$ Equation S12

Subsequently, the volume of the unit cell (V_{cell}), the volume of the S sphere (V_{sphere}), and the radius of the S spheres can be calculated as:

$$V_{cell} = h_{S/VBMImTFSI} \times a_{hex} \times \sqrt{\frac{a \cdot \sqrt{3}}{2}} \quad \text{Equation S13}$$

$$V_{sphere} = V_{cell} \times \frac{\phi_S}{(\phi_{VBMImTFSI} + \phi_S)} \quad \text{Equation S14}$$

$$R_S = \sqrt[3]{\frac{3V_{Sphere}}{4\pi}} \quad \text{Equation S15}$$

where $a_{hex\ lattice}$ is the lattice parameters for the primitive unit cell (trapezoidal prism) of hexagonally packed spheres obtained from SAXS.

As a result, the size of ion transport channels in the L_{SI} morphology (d_4 and d_5 as indicated in Figure S6(B)) can be calculated as:

$$d_4 = a_{hex} - R_S - R_S \quad \text{Equation S16}$$

$$d_5 = h_{S/VBMImTFSI} - R_S - R_S \quad \text{Equation S17}$$

References

1. Cao, Y. C.; Wang, X.; Mamlouk, M.; Scott, K., Preparation of alkaline anion exchange polymer membrane from methylated melamine grafted poly(vinylbenzyl chloride) and its fuel cell performance. *J Mater Chem* **2011,** *21* (34), 12910-12916.
2. Bicak, N.; Ozlem, M., Graft copolymerization of butyl acrylate and 2-ethyl hexyl acrylate from labile chlorines of poly(vinyl chloride) by atom transfer radical polymerization. *J Polym Sci Pol Chem* **2003,** *41* (21), 3457-3462.
3. Cortez-Lemus, N. A.; Salgado-Rodríguez, R.; Licea-Claveríe, A., Preparation of α,ω-telechelic hexyl acrylate polymers with □OH, □COOH, and □NH2 functional groups by RAFT. *Journal of Polymer Science Part A: Polymer Chemistry* **2010,** *48* (14), 3033-3051.
4. Kiefer, J.; Fries, J.; Leipertz, A., Experimental Vibrational Study of Imidazolium-Based Ionic Liquids: Raman and Infrared Spectra of 1-Ethyl-3-methylimidazolium Bis(Trifluoromethylsulfonyl)imide and 1-Ethyl-3-methylimidazolium Ethylsulfate. *Applied Spectroscopy* **2007,** *61* (12), 1306-1311.
5. Meek, K. M.; Sharick, S.; Ye, Y. S.; Winey, K. I.; Elabd, Y. A., Bromide and Hydroxide Conductivity-Morphology Relationships in Polymerized Ionic Liquid Block Copolymers. *Macromolecules* **2015,** *48* (14), 4850-4862.
6. Patnode, W.; Scheiber, W. J., The Density, Thermal Expansion, Vapor Pressure, and Refractive Index of Styrene, and the Density and Thermal Expansion of Polystyrene. *J Am Chem Soc* **1939,** *61* (12), 3449-3451.
7. Ye, C. F.; Shreeve, J. M., Rapid and accurate estimation of densities of room-temperature ionic liquids and salts. *J Phys Chem A* **2007,** *111* (8), 1456-1461.
8. Kimishima, K.; Koga, T.; Hashimoto, T., Order-order phase transition between spherical and cylindrical microdomain structures of block copolymer. I. Mechanism of the transition. *Macromolecules* **2000,** *33* (3), 968-977.
9. Epps, T. H.; Cochran, E. W.; Bailey, T. S.; Waletzko, R. S.; Hardy, C. M.; Bates, F. S., Ordered Network Phases in Linear Poly(isoprene-b-styrene-b-ethylene oxide) Triblock Copolymers. *Macromolecules* **2004,** *37* (22), 8325-8341.
10. Hajduk, D. A.; Harper, P. E.; Gruner, S. M.; Honeker, C. C.; Kim, G.; Thomas, E. L.; Fetters, L. J., The Gyroid - a New Equilibrium Morphology in Weakly Segregated Diblock Copolymers. *Macromolecules* **1994,** *27* (15), 4063-4075.
11. David, J. L.; Gido, S. P.; Hong, K. L.; Zhou, J.; Mays, J. W.; Tan, N. B., Core-shell cylinder morphology in poly(styrene-b-1,3-cyclohexadiene) diblock copolymers. *Macromolecules* **1999,** *32* (10), 3216-3226.
